# A sign-inverted receptive field of inhibitory interneurons provides a pathway for ON-OFF interactions in the retina

**DOI:** 10.1038/s41467-023-41638-3

**Published:** 2023-09-23

**Authors:** Andrew Jo, Sercan Deniz, Jian Xu, Robert M. Duvoisin, Steven H. DeVries, Yongling Zhu

**Affiliations:** 1 Departments of Ophthalmology and Neuroscience, Feinberg School of Medicine, Northwestern University, Chicago, IL 60611 USA; 2 Department of Chemical Physiology and Biochemistry, Oregon Health & Science University, Portland, OR 97239 USA

**Keywords:** Sensory processing, Neural circuits, Retina

## Abstract

A fundamental organizing plan of the retina is that visual information is divided into ON and OFF streams that are processed in separate layers. This functional dichotomy originates in the ON and OFF bipolar cells, which then make excitatory glutamatergic synapses onto amacrine and ganglion cells in the inner plexiform layer. We have identified an amacrine cell (AC), the sign-inverting (SI) AC, that challenges this fundamental plan. The glycinergic, ON-stratifying SI-AC has OFF light responses. In opposition to the classical wiring diagrams, it receives inhibitory inputs from glutamatergic ON bipolar cells at mGluR8 synapses, and excitatory inputs from an OFF wide-field AC at electrical synapses. This “inhibitory ON center - excitatory OFF surround” receptive-field of the SI-AC allows it to use monostratified dendrites to conduct crossover inhibition and push-pull activation to enhance light detection by ACs and RGCs in the dark and feature discrimination in the light.

## Abstract (web summary)

In the retina, visual information is segregated into ON (light increment) and OFF (light decrement) streams. Here, the authors identify a sign-inverting amacrine cell with an inhibitory ON center – excitatory OFF surround receptive field.

## Introduction

The segregation of the visual input into ON and OFF channels that carry information about light increments and decrements, respectively, is a central organizing principle of the vertebrate retina^1–3^. During daylight vision, this segregation begins at the cone synapse where signals diverge to ON and OFF bipolar cells. OFF bipolar cells express ionotropic glutamate receptors and receive a “sign-conserving” input from cones^4–6^. Both cones and OFF bipolar cells depolarize during light decrements. ON bipolar cells express a metabotropic glutamate receptor, mGluR6, that mediates an inversion of the cone signal^7,8^. During light increments, when cones are hyperpolarized, the ON bipolar cells are depolarized. ON and OFF pathway segregation continues in the inner plexiform layer (IPL) where the axon terminals of ON bipolar cells end in the proximal half or sublamina *b* while the terminals of OFF bipolar cells end in the distal half or sublamina *a*. ON and OFF bipolar cells both release glutamate onto excitatory, ionotropic glutamate receptors expressed by ACs and retinal ganglion cells (RGCs), which inherit “ON” and/or “OFF” response polarities depending on their IPL stratifications^2,9–16^. A minor exception in the mammalian retina is that some ON bipolar cells make excitatory ribbon synapses en passant at the top of sublamina *a*, endowing a few AC and RGC types with ON responses^17^. However, even in this case, the excitatory output of the ON bipolar cell determines postsynaptic response polarity. The division of the IPL into ON and OFF sublamina based on the excitatory glutamatergic output of bipolar cell axon terminals is a simplifying principle that is frequently used to predict the responses of AC and RGCs based on their dendritic stratifications alone.

Parallel ON and OFF pathways are not isolated visual channels but instead interact with each other in a process called crossover inhibition. In crossover inhibition, excitation in either the ON or OFF pathway produces an inhibition in local OFF or ON pathways, respectively. Crossover inhibition is mediated by specific ACs with processes that span the OFF and ON sublaminas^18,19^. It has been shown that crossover inhibition extends the range for light responses in some RGC types^20^, compensates for the distorting effects of synaptic rectification^18^, and generates sustained signals in the inner retina^21^. Identifying the subset of ACs that mediate crossover inhibition is a challenge since at least 60 types of ACs are revealed by single-cell RNA sequencing^22,23^, along with 40-50 AC types that have been described in morphological studies^24–26^. It is believed that each AC type has a distinct functional role in visual processing, yet the correspondence is only known for fewer than 20 types^27–40^.

Here we use a Cre/tTA intersectional strategy^41,42^ that relies on two orthogonal binary systems, Cre/loxP and tTA/TRE^43^, to restrict AC labeling and provide genetic access to individual cell types for functional studies. By creating a VGAT-iCreER;Scg2-tTA intersection, we identified an AC type, named SI-AC (sign-inverting AC), whose response properties challenge the classical circuit diagrams of the retina. SI-AC stratifies exclusively in the ON sublamina of the IPL but generates an OFF response, *i.e*. a hyperpolarization to a receptive field center light stimulus and a depolarization to darkness in the receptive field surround, in opposition to expectation. The central OFF response of SI-AC is mediated by a sign-inverting metabotropic receptor (mGluR8) at synapses with ON bipolar cells, while the OFF-surround activation is communicated to SI-AC via electrical synapses from a wide-field AC that receives a typical, sign-preserving input from OFF bipolar cells. Continuously depolarized in the dark, the SI-AC provides crossover inhibition to the ACs and RGCs whose dendrites costratify in the ON sublamina. When hyperpolarized by a central light, the crossover inhibtion is relieved, enhancing the output signals of the ON pathways.

## Results

### SI-AC identified by using a VGAT-iCreER/Scg2-tTA intersectional approach

**Fig. 1:**
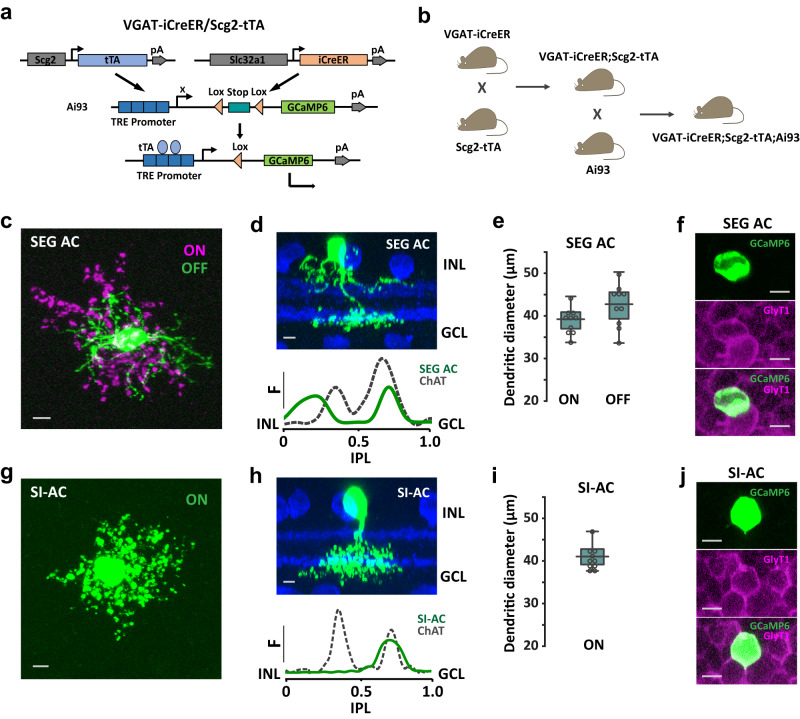
Two new AC types identified in VGAT-iCreER;Scg2-tTA;Ai93 mice. **a** Schematic representation of the VGAT-iCreER/Scg2-tTA intersectional strategy for labeling ACs in the Scg2-tTA driver. The reporter/effector, here GCaMP6f, is only activated in cells expressing both Cre and tTA. **b** Triple transgenic mouse breeding scheme for labeling the VGAT-iCreER/Scg2-tTA intersection. **c**–**j** SEG AC and SI-AC were identified after applying Tamoxifen in adults (8–12 weeks). **c** Collapsed confocal stack of a flatmount view of SEG AC showing processes that ramify in the ON (red) and OFF (green) IPL sublaminas. Scale bar, 5 µm. **d** Transversal view of SEG AC (top), with the dendritic tree fluorescence profile across the IPL (bottom; 0 = INL, 1.0 = GCL). ChAT-positive (blue) bands serve as fiducial markers. INL: the inner nuclear layer. GCL: the ganglion cell layer. IPL: the inner plexiform layer. Scale bar, 5 µm. **e** Dendritic tree diameters of SEG AC in the ON- and OFF-sublaminas (average of 10 cells, error bars: SEM). **f** A GCaMP6f-positive SEG AC colocalized with labeling for the GlyT1 glycine transporter. Scale bars, 5 µm. **g** Collapsed confocal stack of a flatmount view of SI-AC showing processes that ramify in the ON IPL sublamina. Scale bar, 5 µm. **h** Transversal view of SI-AC (top) and dendritic profile (bottom). ChAT: blue. Scale bar, 5 µm. **i** Dendritic tree diameter of SI-AC in the ON-sublamina (average of 10 cells, error bars: SEM). **j** A GCaMP6f-positive SI-AC colocalized with labeling for the GlyT1 glycine transporter. Scale bars, 5 µm. **d**, **h** F: fluorescence. **e**, **i** The box plots display the mean, 25th, and 75th percentiles, while the whiskers indicate the 1.5 interquartile range. Source data are provided as a Source Data file. **c**, **d**, **f**–**h**, **j** Experiments were replicated independently in at least 30 cells with similar results.

The Scg2-tTA driver expresses tTA under the control of a mouse secretogranin II promoter^44^. In the retina, tTA was expressed in multiple types of ACs and RGCs. To isolate tTA-expressing ACs and to label them sparsely for analysis, we crossed Scg2-tTA mice with VGAT-iCreER (an inducible Cre driver under the control of Slc32a1 promotor) mice and used a Cre/tTA dependent-GCaMP6f reporter line Ai93^42^ to label cells in the intersection (Fig. 1a, b). After applying Tamoxifen in adults (8–12 weeks), two types of narrow-field ACs were labeled together with sparsely labeled wide-field ACs. The first type resembles the previously described SEG (Satb2^+^Ebf3^+^Glyt1^+^) AC^45^ while the second type is named SI-AC based on functional characteristics to be described later on. SEG AC and SI-AC occurred in a ratio of ~3:2. The soma of SEG AC was in the inner nuclear layer (INL) and its dendrites ramified in two bands: one adjacent to the ON ChAT band (0.60 ± 0.03–0.77 ± 0.03 of the IPL, *n* = 10) and the other between the OFF ChAT band and the INL (0.05 ± 0.02–0.26 ± 0.01 of the IPL, *n* = 10) (Fig. 1c, d). The dendritic field diameters of SEG AC were 39.2 ± 1.0 μm (ON, *n* = 10) and 42.7 ± 1.5 μm (OFF, *n* = 10) (Fig. 1e), and the cell was glycinergic based on GlyT1 immunoreactivity (Fig. 1f). The soma of SI-AC was also located in the INL and its dendrites were confined to the ON sublamina (0.50 ± 0.01–0.82 ± 0.04 of the IPL, *n* = 10) (Fig. 1g, h). The dendritic diameter of the SI-AC was 41.0 ± 0.9 μm (*n* = 10) (Fig. 1i) and the cell was glycinergic (Fig. 1j). The different stratification patterns allowed us to unambiguously distinguish SI-AC from SEG AC for further study. Morphological features suggest that SEG AC resembles AC type 18, while SI-AC resembles type 22 or possibly type 23 in a previous serial block-face electron microscopy study^25,46^.

### SI-AC exhibits light responses as opposed to prediction

**Fig. 2:**
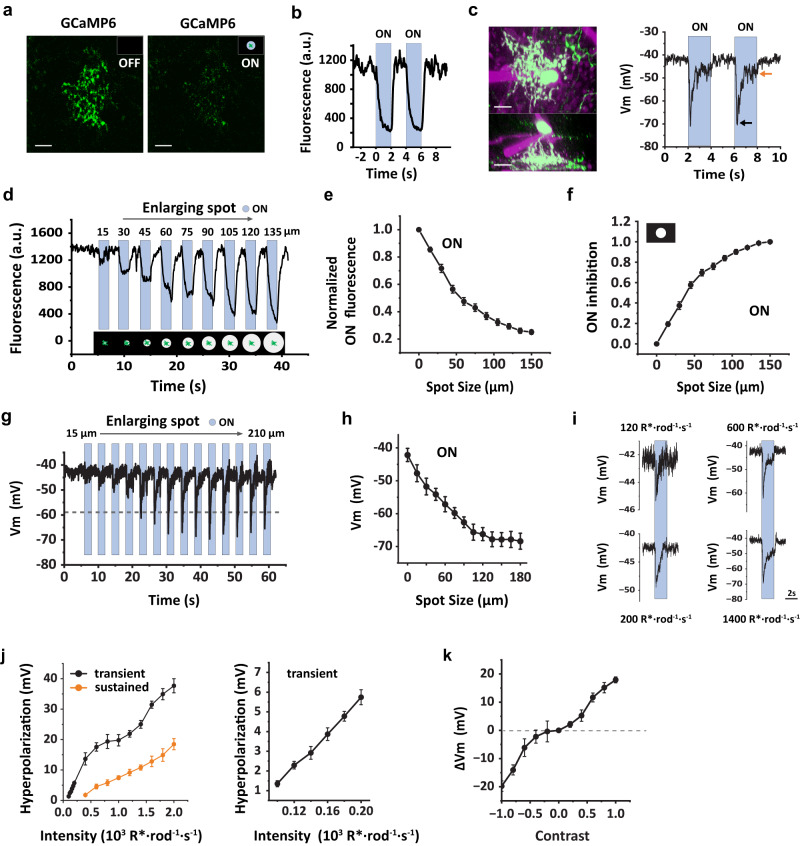
SI-AC was activated in the dark and inhibited by the light. **a**, **b** Two-photon GCaMP6f fluorescence in SI-AC dendrites was high in the dark (OFF) but decreased when stimulated a spot of light (100 µm diameter, 100% contrast) was presented in the center of the receptive field. Blue bars represent light presentation. Scale bars, 10 µm. Experiments were replicated independently in at least 40 cells with similar results. **c** SI-AC was targeted for whole-cell recording (left upper panel, flat-mount view, lower panel, side view). Membrane potential was depolarized in the dark and hyperpolarized by light (100 µm dia. spot, right). Black arrow: transient hyperpolarization. Orange arrow: sustained hyperpolarization. Scale bars, 10 µm. Experiments were replicated independently in at least 30 cells with similar results. **d** GCaMP6f fluorescence as a function of light spot diameter. Larger spots cause larger fluorescence decreases (100% contrast on a dark background, SI-AC dendritic field indicated in green, illustrated below). **e** Decrease in normalized fluorescence as a function of spot diameter (average of 27 cells, error bars: SEM). **f** Spatial profile of ON inhibition calculated from **e**. ON inhibition = (*F*_0_−*F*)/(*F*_0_−*F*_150_) where the subscript denotes spot diameter. Error bars: SEM. **g** Whole-cell recording of membrane potential as a function of light spot diameter. **h** Plot of peak response as a function of spot diameter (average of 9 cells, error bars: SEM). **i** Whole-cell recording of membrane potential in response to a light spot (100 µm) at different intensity. **j** Left: plot of light-evoked transient and sustained hyperpolarization as a function of intensity (average 5 cells, error bar: SEM). The boxed region is enlarged on the right. **k** Contrast sensitivity at the background light level (1000 R*/rod/s) for whole-cell recording (average 5 cells, error bar: SEM). Experiments were performed in VGAT-iCreER;Scg2-tTA;Ai93 mice.

We next examined the light responses of SI-AC. Since SI-AC’s dendrites broadly stratified in the ON sublamina, we predicted that it would receive direct excitatory inputs from ON bipolar cells and act as an “ON” cell. Against expectation, two-photon imaging of dendrites showed that GCaMP6f fluorescence remained elevated in the nominal dark (two-photon scanning produces an effective background of ~1000 R*/rod/s, Supplementary Fig. 1), suggesting that dendrites are depolarized, and decreased rapidly when a spot of light (100 µm diameter) with an equivalent intensity of ~1500 R*/rod/s was applied in the receptive field center (Fig. 2a, b). To verify that SI-ACs were depolarized in the dark, we measured changes in membrane potential in response to the light stimulus using a whole-cell current-clamp. In these experiments, two-photon illumination was present while locating the GCaMP6f-expressing SI-AC but absent during the subsequent whole-cell recording. In the dark, SI-AC rested at −42.1 ± 2.0 mV (*n* = 9). A spot of light (100 µm diameter, ~1500 R*/rod/s) in the receptive field center produced a transient hyperpolarization to −66.6 ± 2.4 mV followed by a decay to a sustained value of −48.3 ± 2.1 mV (*n* = 9, Fig. 2c). Thus, instead of behaving as an “ON” AC, SI-AC behaved as an “OFF” type that was activated (i.e., depolarized) in the dark but inhibited (hyperpolarized) by light. Differences in the time course of the GCaMP6f and membrane potential responses likely result from a limited voltage range of membrane Ca^2+^ channel activation. Differences in the steady background illumination may also contribute (Supplementary Fig. 1).

To map the receptive field center of SI-AC, we applied a series of expanding, concentric spots. We found that the GCaMP6f signal quickly decreased with enlarging light spots on a dark background (Fig. 2d, e). The “light-ON inhibition” reached ~76% of the maximum with a 75 µm diameter spot (Fig. 2f). The spatial profile of membrane potential hyperpolarization was similar (Fig. 2g, h). These results suggest that the mechanism of light-induced hyperpolarization resides in the receptive field center.

We next examined the intensity-response properties of SI-AC by applying a 100 µm spot of light that was stepped from complete darkness to different intensity levels (Fig. 2i). As shown in Fig. 2j, transient voltage responses increased linearly up to stimulus intensities of 200 R*/rod/s (*n* = 5). Subsequently, the responses exhibited a sublinear pattern until they reached 1000 R*/rod/s. Notably, at higher photopic levels, another phase of increase was observed. Since we had to target GCaMP6f-expressing SI-ACs through two-photon imaging for whole-cell recording, a persistent adaptation limited our ability to obtain responses to very dim flashes (below 100 R*/rod/s). The contrast sensitivity during exposure to background light at low photopic levels (1000 R*/rod/s) is shown in Fig. 2k.

### SI-AC receives glutamatergic inhibition from the ON pathway

We first considered whether ON hyperpolarization may result from disynaptic disinhibition involving a chain of synapses involving an ON bipolar cell, an ON AC, and the SI-AC. To test this possibility, we added gabazine, a selective GABA_A_ antagonist, and strychnine, a selective glycine receptor antagonist, to the bath and measured ON hyperpolarization with GCaMP6f imaging. As shown in Supplementary Fig. 2a, strychnine had no effect on the ON response. Gabazine reduced the ON inhibition by 32%, but only had an effect on responses to spots ≥100 µm in diameter (Supplementary Fig. 2b). These results suggest that the center response to a small spot is produced by a non-glycinergic and non-GABAergic mechanism. The effect of gabazine on responses to spots ≥100 µm is consistent with a GABAergic component of the receptive field surround.

#### SI-AC receives glutamatergic inputs from ON bipolar cells

**Fig. 3:**
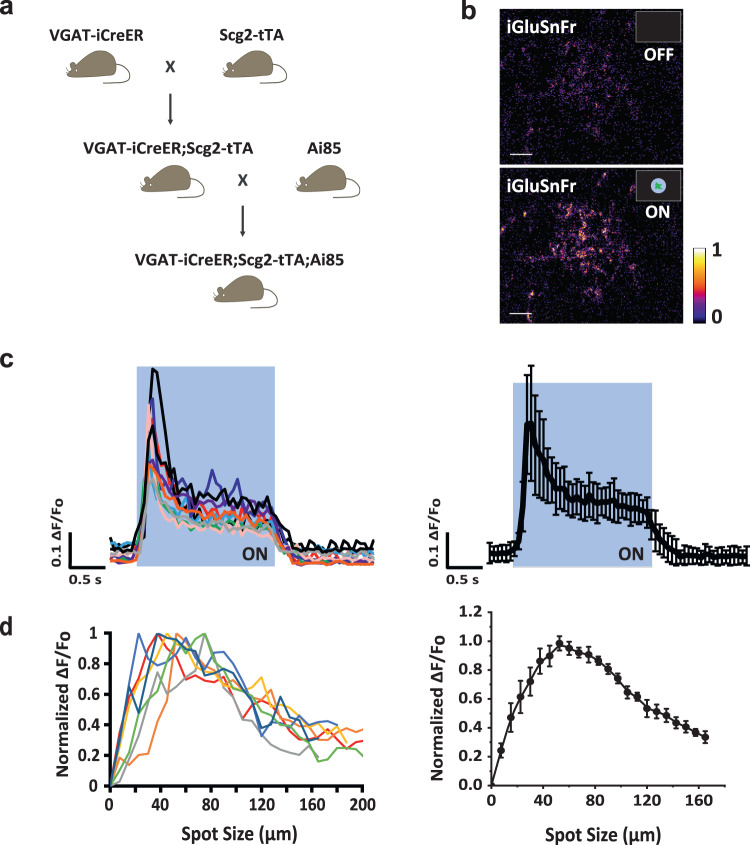
SI-AC received direct glutamatergic inputs from ON bipolar cells. **a** Triple transgenic breeding scheme for expressing iGluSnFr in SI-AC. Ai85 is a Cre/tTA dependent-iGluSnFr reporter mouse line. **b** Two-photon iGluSnFr fluorescence in SI-AC dendrites in the dark (OFF) and when a spot of light (60 µm, 100% contrast) was applied to the center of a black background (0% contrast) (ON). Scale bar, 10 µm. Response is presented using min-max normalization, the color bar corresponds to a range of 0 to 1. Experiments were replicated independently in at least 20 cells with similar results. **c** Left: iGluSnFr spot responses for 10 cells shown in different colors. Right: average response (error bars: SD). **d** Left: Individual spatial profiles of seven cells (shown in different colors). Right: Averaged spatial profile (error bars: SEM). Fluorescence values were the averages across the dendritic field.

Although glutamate acts as an excitatory neurotransmitter at bipolar cell synapses, we considered the possibility that glutamate could have an inhibitory action if postsynaptic neurons use group II or III mGluRs to mediate transmission. To test this possibility, we first confirmed that SI-AC received direct glutamatergic input from ON bipolar cells. We delivered the glutamate sensor iGluSnFr to SI-AC with Ai85, a Cre/tTA dependent-iGluSnFr reporter line^42^ (Fig. 3a). Two-photon imaging revealed that a small spot of light (60 µm) evoked iGluSnFr responses in the dendrites (Fig. 3b, c). The spatial profile of iGluSnFr responses showed a peak at around 50 µm (Fig. 3d), in agreement with the size of the dendritic field of SI-AC (Fig. 1i). These results demonstrate that SI-AC receives direct glutamatergic inputs from ON bipolar cells. The reduction in iGluSnFr responses during spots larger than 50 µm is consistent with a presynaptic inhibition of the bipolar cells from other ACs (Fig. 3d).

#### mGluR8 mediates light-evoked center inhibition

**Fig. 4:**
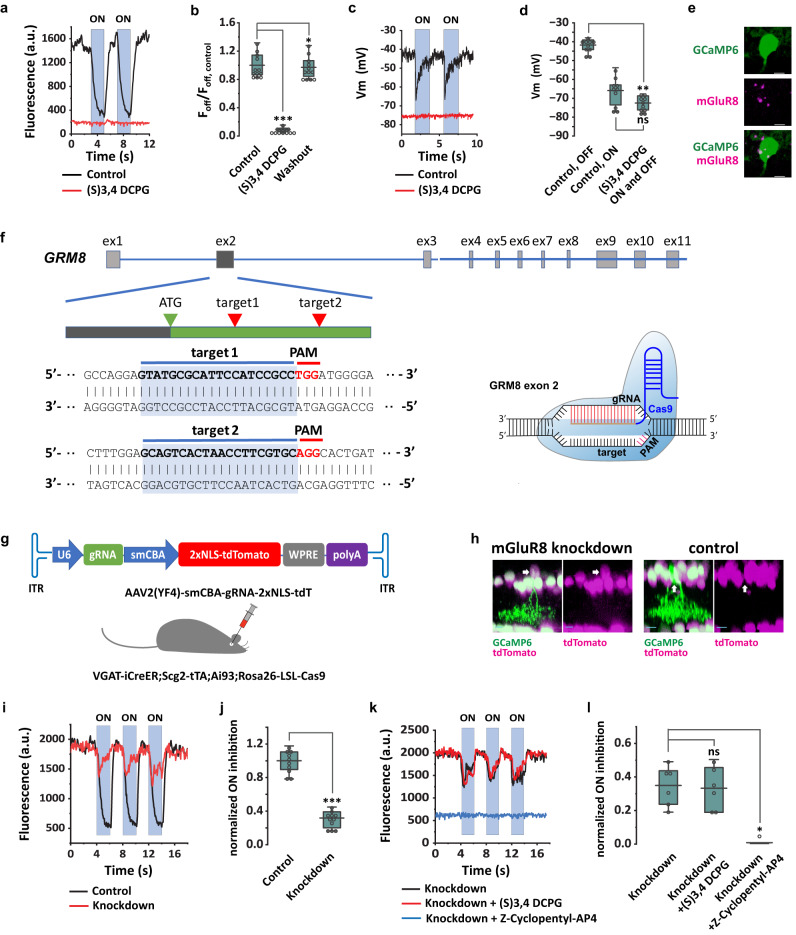
ON inhibition in SI-AC is mediated by mGluR8. **a** (S) 3,4 DCPG (0.5 µM), a selective mGluR8 agonist, abolished GCaMP6f responses in the dark and light. **b** Effect of (S) 3,4 DCPG on the GCaMP6f signal in the dark, *n* = 12 cells for all. ***p = 4.8E-4, *p = 0.016, Wilcoxon Signed Rank test, two-tailed. **c** (S) 3,4 DCPG hyperpolarized the membrane potential and blocked the ON response. **d** Effect of (S) 3,4 DCPG on the membrane potential (*n* = 10 cells), **p = 0.0020, ns: *p* = 0.064, Wilcoxon Signed Rank test, two-tailed. **e** In situ hybridization confirmed the mGluR8 expression in SI-AC. Scale bar, 5 µm. **f** CRISPR design for mGluR8 knockdown. Exon2 of GRM8 gene contained the guide RNA (gRNA) targets. **g** AAV2(YF4) encoding Grm8 exon2 gRNA and the 2xNLS-tdTomato nuclear marker (top) was injected into Cas9 mice. **h** SI-AC with mGluR8 knockdown expressed both GCaMP6 and tdTomato (arrow), while control SI-AC only expressed GCaMP6f. Scale bar: 10 µm. **i** GCaMP6f responses in control (black) and mGluR8 knockdown (red) cells. **j** Effect of mGluR8 knockdown on the ON inhibition, *n* = 10 cells for control, *n* = 11 cells for knockdown. ON inhibition = (F_OFF_−*F*_ON_)/*F*_OFF_, normalized to control. ***p = 1.2E-4, Mann-Whitney Test, two-tailed. **k** GCaMP6f responses in mGluR8 knockdown were not affected by (*S*)−3,4-DCPG but were blocked by Z-Cyclopentyl-AP4 (50 µM), *n* = 6 cells for all. **l** Effect of 3,4 DCPG and Z-Cyclopentyl-AP4 on the ON inhibition (normalized to control) in mGluR8 knockdown, *n* = 6 cells for all. ns: *p* = 0.63, *p = 0.031, Wilcoxon Signed Rank test, two-tailed. **a**, **c**, **i**, **k** ON responses were evoked with 60 µm light spot. **b**, **d**, **j**, **l** the box plots display the mean, 25th, and 75th percentiles, while the whiskers indicate the 1.5 interquartile range. Source data are provided as a Source Data file. **a**–**e** were performed on VGAT-iCreER;Scg2-tTA;Ai93 mice. **f**–**l** were performed on VGAT-iCreER;Scg2-tTA;Ai93;Rosa26-LSL-Cas9 mice. Experiments were replicated independently in at least 20 cells for **e** and in at least 15 cells for **h** with similar results.

The iGluSnFr results associate glutamate release onto SI-AC dendrites with a membrane hyperpolarization in SI-AC. This inhibitory or sign-inverting synaptic response could be mediated by metabotropic glutamate receptors. To investigate whether group III mGluRs were involved in the ON inhibition, we applied the group III mGluRs selective agonist L-AP4 in the bath. In the absence of group III receptors on SI-AC, the expectation is that L-AP4, which hyperpolarizes ON bipolar cells by acting at mGluR6 receptors on dendrites in the outerplexiform layer, should block the glutamate release onto SI-AC and relieve the membrane hyperpolarization. However, L-AP4, at a concentration of 1 µM, produced a decrease in the intracellular Ca^2+^ concentrations that persisted during both darkness and light (Supplementary Fig. 3a), suggesting a sustained hyperpolarization. These results suggested that L-AP4 directly hyperpolarized SI-AC through group III mGluRs. Group III mGluRs encompass four members: mGluR4, mGluR6, mGluR7, and mGluR8^47^. To determine which mGluR(s) were responsible for the ON inhibition, we examined the function of each group III mGluR by adding specific agonists or modulators to the bath. We found that (*S*)-3,4-DCPG, a specific agonist for mGluR8 (EC_50_ = 31 nM for mGluR8)^48,49^, abolished GCaMP6f responses in the dark at a concentration of 0.5 µM (Fig. 4a, b). Whole-cell recording showed that 0.5 µM (*S*)-3,4-DCPG hyperpolarized the SI-AC membrane potential to −72.5 ± 1.4 mV (*n* = 10) in both dark and light (Fig. 4c, d). To confirm mGluR8 expression, we performed in situ hybridization in GCaMP6f labeled SI-AC and found colocalization with mGluR8 expression (Fig. 4e). On the other hand, agonists that are more selective for other Group III mGluRs (Z-Cyclopentyl-AP4 for mGluR4^50^, AMN-082 for mGluR7^51^) and a negative allosteric modulator for mGluR7 (ADX 71743)^52^ had no effect on SI-AC responses (Supplementary Fig. 3b–d). These results strongly suggest that mGluR8 mediates the light-ON hyperpolarization of SI-ACs.

To confirm a necessary role for mGluR8 in the SI-AC inhibitory response, we used CRISPR technology to knockdown mGluR8 in these cells. We constructed a pAAV-U6-sgRNA-smCBA-NLS-tdTomato vector in which a gRNA was used to target the second exon of GRM8 and nuclear-localized tdTomato was used to label the transfected cells (Fig. 4f, g). AAVs were packaged with AAV2(YF4) capsid^53,54^ to enhance the transfection of ACs. We injected AAVs intravitreally into the eyes of Cre-dependent Cas9 mice (Rosa26-LSL-Cas9) that had been crossed with VGAT-iCreER;Scg2-tTA;Ai93 mice (Fig. 4g). SI-ACs infected with AAVs expressed tdTomato in the nucleus and GCaMP6f in the soma and dendrites, while non-infected SI-ACs only expressed GCaMP6f without tdTomato (Fig. 4h). We imaged GCaMP6f responses in both AAV infected (knockdown) and non-infected (control) SI-ACs (Fig. 4i). We found that knocking down mGluR8 decreased the ON inhibition in response to a spot of light to 31.8% ± 3.0% (Fig. 4j). Interestingly, the remaining ON inhibition was not affected by (*S*)-3,4-DCPG, but was instead blocked by Z-Cyclopentyl-AP4, a Group III mGluR antagonist that is more selective for mGluR4 (Fig. 4k, l). The results are consistent with a scenario in which CRISPR knockdown completely suppressed mGluR8 expression in transfected cells, in turn triggering a compensatory upregulation of mGluR4. Therefore, we concluded that mGluR8 mediates light-ON center hyperpolarization in SI-ACs.

#### Light-evoked inhibition is mediated by Gβγ and GIRK channel

**Fig. 5:**
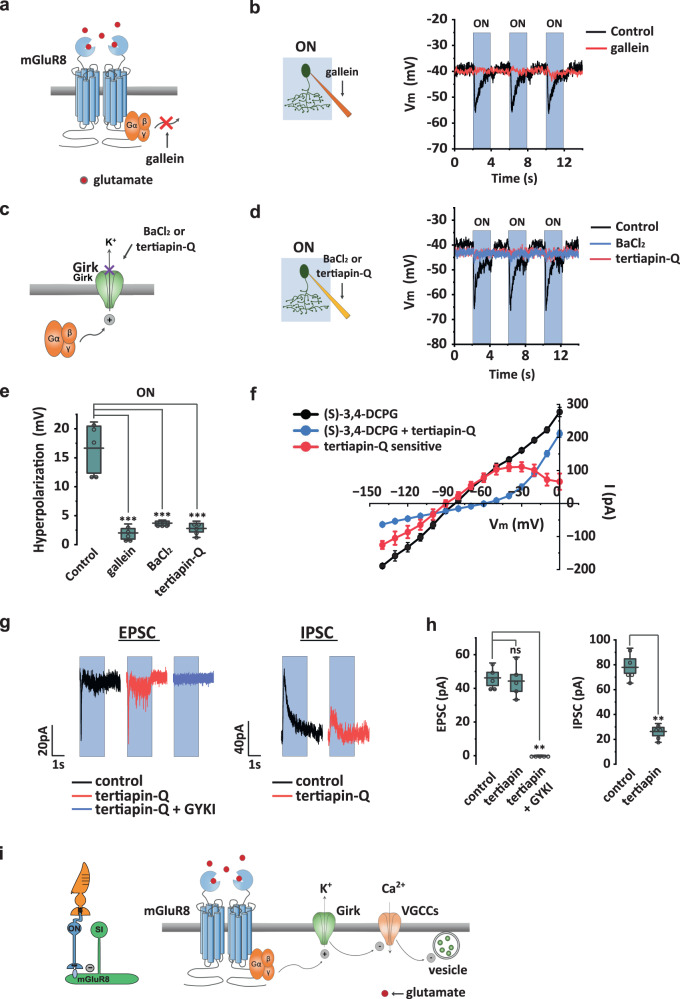
ON hyperpolarization in SI-AC is mediated by mGluR8 via G protein βγ subunits and GIRK channels. **a** Diagram showing gallein block of G protein βγ (Gβγ) subunits signaling. **b** Intracellular gallein (10 µM in the pipette solution) abolished light-evoked hyperpolarization. **c** BaCl_2_ and tertiapin-Q act intracellularly to block GIRK channels. **d** BaCl_2_ (10 µM) or tertiapin-Q (100 nM) in the pipette solution abolished light-evoked membrane hyperpolarization. **e** Summary of inhibition by gallein, BaCl_2_, and tertiapin-Q on light-evoked hyperpolarization. *N* = 5 cells for all. **p = 0.0061 for all, Mann–Whitney test, one-tailed. **f** I–V curve for the tertiapin-Q sensitive current in (*S*)−3,4-DCPG (red) obtained by subtracting the curve in (*S*)−3,4-DCPG + tertiapin-Q (green) from the (*S*)−3,4-DCPG curve (black) (*n* = 5 cells). Error Bars: SEM. **g** Left: light-evoked EPSC measured at −90 mV was completely blocked by GYKI 53655 (25 µM). Right: light-evoked IPSC measured at 0 mV was inhibited by tertiapin-Q. ON responses were evoked with a 100 µm light spot. **h** Summary of effects of GYKI 53655 in EPSC and tertiapin-Q in IPSC (*n* = 5 cells). EPSC: ns: *p* = 0.42, **p = 0.0061, Mann–Whitney Test, one-tailed. IPSC: **p = 0.0061, Mann–Whitney test, one-tailed. **i** Model of ON inhibition. Left: light triggers glutamate release from ON bipolar cells. Right: glutamate binding to mGluR8 activates GIRK channels via Gβγ subunits, leading to membrane hyperpolarization and subsequent inhibition of voltage-gated calcium channels (VGCCs) and cessation of glycine release in SI-AC. ON responses in **b**, **d**, **g** were evoked with a 60 µm light spot. Coupling between SI-AC and other cells was blocked in F with MFA (25 µM) to improve the voltage clamp. **e**, **h** The box plots display the mean, 25th, and 75th percentiles, while the whiskers indicate the 1.5 interquartile range. Source data are provided as a Source Data file. All experiments were performed on VGAT-iCreER;Scg2-tTA;Ai93 mice.

mGluR8 is a Gi/o protein-coupled receptor. To examine a role for Gβγ in the SI-AC response, we added gallein, a Gβγ signaling inhibitor (Fig. 5a) to the pipette solution and measured membrane potential with current-clamp recording (Fig. 5b). 10 µM gallein in the pipette blocked 93.7% ± 10.3% (n = 6) of the hyperpolarization during light without affecting the membrane potential in the dark (Fig. 5b, e, and Supplementary Fig. 4). This result suggests that the majority of the hyperpolarization triggered by light is mediated by Gβγ.

What could be the downstream target of Gβγ that leads to the hyperpolarization? G protein-gated inwardly rectifying K^+^ (GIRK) channels are often direct targets of Gβγ activity^55,56^. We examined the role of GIRK channels by using two selective blockers (Fig. 5c): BaCl_2_ at sub-millimolar concentrations^57–59^ and tertiapin-Q^60–62^. We added 10 µM BaCl_2_ or 100 nM tertiapin-Q to the pipette solution and measured SI-AC membrane potential with current-clamp recording. Both agents effectively blocked hyperpolarization during light stimulation without changing membrane potential in the dark (Fig. 5d and Supplementary Fig. 4). BaCl_2_ blocked 82.2% ± 9.2% (*n* = 6) of the hyperpolarization and tertiapin-Q blocked 88.5% ± 7.5% (*n* = 6) of the hyperpolarization (Fig. 5d, e). Consistent with their effects on the light response, gallein, BaCl_2_, and tertiapin-Q in the bath also reversed the effect of (*S*)-3,4-DCPG on the GCaMP6 signal when applied during darkness (Supplementary Fig. 5).

Gβγ activation has been linked both to the activation of K^+^ channels^55^ and the inhibition of Ca^2+^ channels^63,64^. In order to provide further evidence that mGluR8 modulates a K^+^-conductance, we measured the I-V curve in (S)-3,4-DCPG with control or tertiapin-Q in the pipette solution (Fig. 5f, black and green). The subtraction of the two I-V curves revealed the tertiapin-Q sensitive component in (S)-3,4-DCPG, which exhibited a reversal potential of −90 mV and significant rectification that began at −50 mV and became stronger after −30 mV (Fig. 5f, red). The I-V curve of the tertiapin-Q sensitive component overlaps with that of (S)-3,4-DCPG between −40 mV (the dark activation level) and −90 mV, suggesting that GIRK channels were responsible for the hyperpolarization induced by (S)-3,4-DCPG. The rectification of the GIRK channels enables them to pass a large potassium current at membrane potentials around or negative to −40 mV while shutting down the potassium current at more positive potentials to allow SI-AC to be activated by other mechanisms. To examine the underlying excitatory and inhibitory currents that mediate the light-ON response, we measured the EPSC (*V*_m_ = −90 mV) and IPSC (*V*_m_ = 0 mV) in whole-cell voltage clampe during steps of a 100 µm light spot (Fig. 5g). Light ON stimulation produced a small and transient EPSC (46.2 ± 2.8pA, decay time constant of 29 ± 1 ms, *n* = 5) and a larger and more sustained IPSC (77.9 ± 7.9 pA, decay time constant of 421 ± 18 ms, *n* = 5). The EPSC was entirely inhibited by 25 µM GYKI 53655 (Fig. 5g, h), indicating that it was mediated by AMPA receptors. The rapid decay constant of the EPSC reflects the rapid desensitization of AMPA receptors. In contrast, tertiapin-Q in the pipette blocked approximately 66% of the IPSC (Fig. 5g, h), suggesting that two-thirds of the IPSC at 0 mV were mediated by GIRK channels. The remaining one-third was likely mediated by GABAergic inhibition at a 100 µm spot of light (as shown in Supplementary Fig. 2b). Given the strong rectification of GIRK channels at −30 mV (Fig. 5f, red), we can expect GIRK channel-mediated inhibition to be even more dominant at membrane potentials negative to −40 mV. Although we cannot rule out the possibility that mGluR8 inhibits Ca^2+^ channels, the lack of a voltage change in the light presence when intracellular tertiapin-Q and BaCl_2_ are present (Fig. 5d, e) at voltages where Ca^2+^ channels are active makes this possibility less likely.

Based on these results, we propose a model for the signaling cascade of SI-AC ON hyperpolarization (Fig. 5i). The cascade begins with the stimulation of mGluR8 by synaptic glutamate released from ON bipolar cells. This triggers the dissociation of Gβγ from Gi/o, leading to the activation of GIRK by Gβγ. The activation of GIRK causes hyperpolarization of the membrane potential, which subsequently inactivates voltage-gated Ca^2+^ channels, reduces intracellular Ca^2+^ concentrations, and inhibits glycine release from SI-AC.

### SI-AC receives excitation from the OFF pathway

**Fig. 6:**
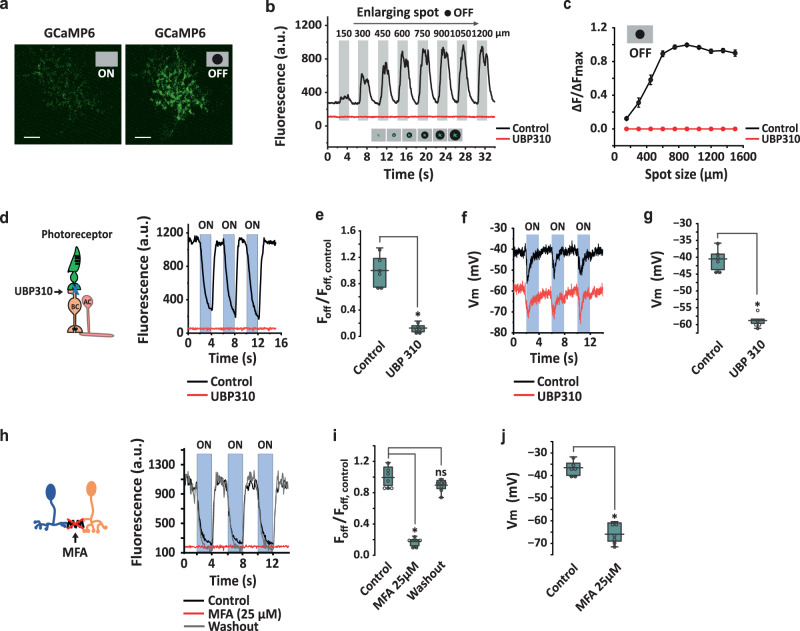
Pharmacological interventions suggest an OFF surround mechanism. **a**, **b** Measurement of light-OFF responses as a function of dark spot (0% contrast) diameter presented on a gray background (50% contrast) in control and 25 µM UBP310. Spot in **a**: 60 µm. Experiments were replicated independently in at least 30 cells with similar results. **c** Spatial profile of OFF responses (*n* = 26 cells) in control and UBP310. ∆*F*/∆*F*max = (*F*−*F*_0µm_)/(*F*_900µm_−*F*_0µm_). Data are reported as mean ± SEM. **d** UBP310 blocked the GCaMP6f signal in the dark and abolished the light response. BC: OFF bipolar cell. AC: amacrine cell. Spot of light (60 µm) applied on a dark background. **e** Summary of UBP310 effect on the GCaMP6f signal in the dark (*n* = 6 cells). *p = 0.031, Wilcoxon Signed Rank test, two-tailed. **f** Whole-cell recording shows that UBP310 shifted the membrane potential and light response to a more hyperpolarized level. **g** Summary of UBP310-induced hyperpolarization in the dark (*n* = 5 cells). *p = 0.031, Wilcoxon Signed Rank test, one-tailed. **h** GCaMP6f fluorescence in the dark was reduced and the light response abolished by the gap junction blocker MFA (25 µM). **i** Summary of MFA inhibition on the GCaMP6f signal in the dark (*n* = 7 cells). *p = 0.016, ns: *p* = 0.11, Wilcoxon signed rank test, two-tailed. **j** Summary of 25 µM MFA-induced hyperpolarization in the dark (*n* = 6 cells). *p = 0.016, Wilcoxon Signed Rank test, one-tailed. **e**, **g**, **i**, **j** The box plots display the mean, 25th, and 75th percentiles, while the whiskers indicate the 1.5 interquartile range. Source data are provided as a Source Data file. All experiments were performed on VGAT-iCreER;Scg2-tTA;Ai93 mice.

To measure the OFF receptive field of SI-AC, we modified our central stimulus and instead applied an expanding dark spot (0% contrast) on a gray background (50% contrast) (Fig. 6a, b). GCaMP6f fluorescence in the SI-AC dendrites maintained its low baseline level for small dark spots <150 µm (Supplementary Fig. 6), but gradually increased with enlargement and reached a peak at a diameter around 750 µm (Fig. 6b, c), suggesting that a component of light-OFF activation comes from the periphery.

#### OFF excitation originates in OFF bipolar cells

To implicate a role for OFF bipolar cells in the OFF activation, we applied UBP310, an OFF bipolar cell kainate receptor antagonist, while presenting a small spot of light on a dark background to an SI-AC cell (Fig. 6d). UBP310 drove the GCaMP6f signal to a uniformly low level both in the dark and during the light stimulus (Fig. 6d, e). Correspondingly, UBP310 blocked GCaMP6f responses for the OFF receptive field measurement (Fig. 6b, c, red). Parallel whole-cell recording showed that the addition of UBP310 to the bath hyperpolarized the SI-AC membrane potential in the dark from −41.4 ± 2.2 mV to −58.8 ± 1.5 mV (*n* = 5, Fig. 6f, g), a level below which sustained Ca^2+^ channel types are normally open. However, even at this relatively hyperpolarized level, the central light inhibitory response was maintained. Since UBP310 mimics the hyperpolarizing effect of light on OFF bipolar cells, the results suggest a mechanism in which the peripheral dark excitation/light inhibition in SI-ACs is transmitted by OFF bipolar cells through a sign-preserving mechanism.

#### OFF excitation is mediated by electrical coupling

How can SI-AC’s dendrites, which ramify in the ON sublamina, receive excitation from OFF bipolar cells which project to the OFF sublamina? One possibility for this “crossover excitation” could involve a bistratified AC that receives excitation from OFF bipolar cells and then conveys the excitation to SI-AC dendrites via “sign-conserving” electrical synapses in the ON sublamina. To examine whether electrical coupling contributes to the OFF excitation, we applied the gap junction blocker MFA (25 µM) in the bath. MFA completely eliminated SI-AC OFF excitation measured with both GCaMP6f (Fig. 6h, i) and whole-cell patch-clamp (Fig. 6j) recordings, suggesting that OFF excitation was mediated by electrical coupling. The effect of MFA was completely reversed upon washout (Fig. 6h, i).

**Fig. 7:**
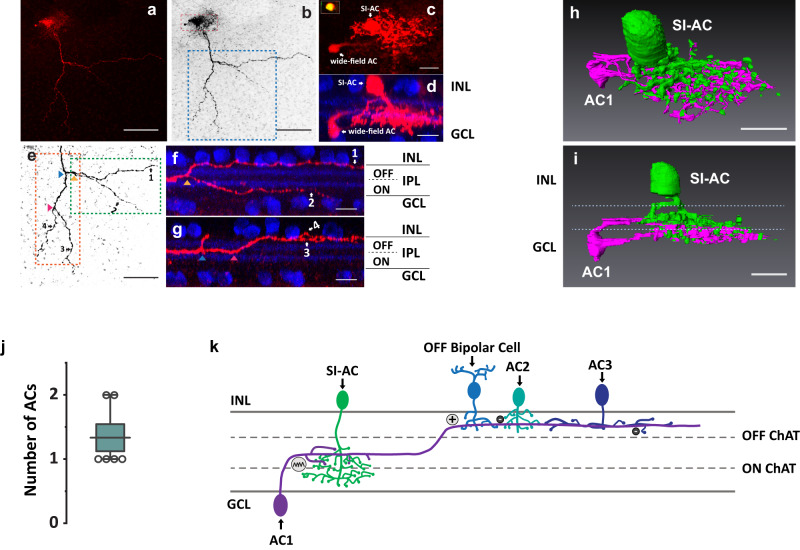
SI-AC is tracer coupled to a wide-field AC. **a** Neurobiotin introduced into SI-AC via a patch-pipette spread to a wide-field AC (red). **b** Grayscale conversion of **a**. Red box is shown enlarged in **c** and blue box is enlarged in **e**. **c** Enlarged view of SI-AC and wide-field AC in whole mount view. Inset: SI-AC soma labeled with GCaMP6f and neurobiotin. **d** Side view of SI-AC and wide-field AC with ChAT immunolabeling (blue). **e** Stratification of wide-field AC neurites. Green box was enlarged and rotated in **f** and orange box was enlarged and rotated in **g**. Arrowheads show branching points. **f**, **g** Transversal view of the primary dendrite of the wide-field AC, which started at the middle of the IPL, then branched and extended (neurites 1, 3, and 4) to the OFF layer between the outer ChAT band (blue) and the INL. **h**, **i** 3D reconstruction of the SI-AC and wide-field AC. SI-AC is pseudo-colored in green, the wide-field AC is in red. Dotted lines in **i**: ON and OFF ChAT bands. **j** Number of neurobiotin-labeled wide-field ACs for a single SI-AC. *N* = 6 SI-ACs. The box plot displays the mean, 25th, and 75th percentiles, while the whiskers indicate the 1.5 interquartile range. Source data are provided as a Source Data file. **k** Model of the OFF activation. AC1 receives excitation from OFF bipolar cells and conveys it to SI-AC in the ON sublamina via electrical synapses. The activity of AC1 is also modulated by glycinergic (AC2) and GABAergic (AC3) ACs. All experiments were performed on VGAT-iCreER;Scg2-tTA;Ai93 mice. Scale bars: 100 μm (**a**, **b**, **e**), 10 μm (**c**, **d**, **f**–**i**). **a**–**d**, **f**, **g** Experiments were replicated independently in at least 6 cells with similar results.

To visualize the cells that are electrically coupled to SI-AC, we recorded from SI-AC with neurobiotin in the patch-pipette solution. Interestingly, neurobiotin labeled a SI-AC along with a wide-field AC (Fig. 7a, b). The soma of the wide-field AC was located in the ganglion cell layer (GCL) and its proximal dendrites contacted SI-AC in the ON sublamina (Fig. 7c, d, h, i). Dendrites of the wide-field AC then traveled between ON and OFF ChAT bands, finally crossing the outer ChAT band to ramify in the OFF sublamina where they could receive excitation from OFF bipolar cells (Fig. 7e–g). The distance between the first ON/OFF crossing point of the wide-field AC dendrites and SI-AC somas was 142.6 ± 40.2 µm (*n* = 6 cells). This result is consistent with the receptive-field measurement showing that the OFF excitation emerged for dark spots larger than an ~150 μm radius (Fig. 6b, c). The number of neurobiotin-labeled wide-field ACs were limited to 1 or 2 in our experiments, with an average of 1.33 ± 0.21 (*n* = 6 cells, Fig. 7j). To verify the specificity of tracer coupling, we added MFA (25 µM) and repeated the neurobiotin labeling. MFA abolished labeling of the wide-field AC and other SI-AC, confirming the specificity of tracer coupling (Supplementary Fig. 7).

#### GABAergic and Glycinergic inhibition on SI-AC OFF response

Thus far our data shows that the OFF excitation in SI-AC originates from a wide-field AC via electrical coupling. We next examined whether SI-AC also receives GABAergic and /or glycinergic inhibition from other ACs in the dark. We applied gabazine (GABA_A_ receptor blocker) and strychnine (glycine receptor blocker) in the bath and measured GCaMP6f responses of SI-AC in the dark. As shown in Supplementary Fig. 8, gabazine slightly increased GCaMP6f signals by 14.8% ± 2.6% (*n* = 7), and strychnine increased GCaMP6 signals by 18.5% ± 2.7% (*n* = 9). Therefore, SI-AC received moderate OFF inhibition from other GABAergic and glycinergic ACs.

Based on these results, we proposed a model for surround OFF excitation of SI-AC (Fig. 7k). In this model, a bistratified wide-filed AC (AC1) receives excitation from a sustained OFF bipolar cell in the OFF sublamina and then makes gap junctional connections with SI-AC in the ON sublamina and activates it in the dark. This OFF excitation is modified by inhibitory inputs from narrow-filed ACs (AC2) and wide-field ACs (AC3).

### Unconventional receptive field of SI-AC

**Fig. 8:**
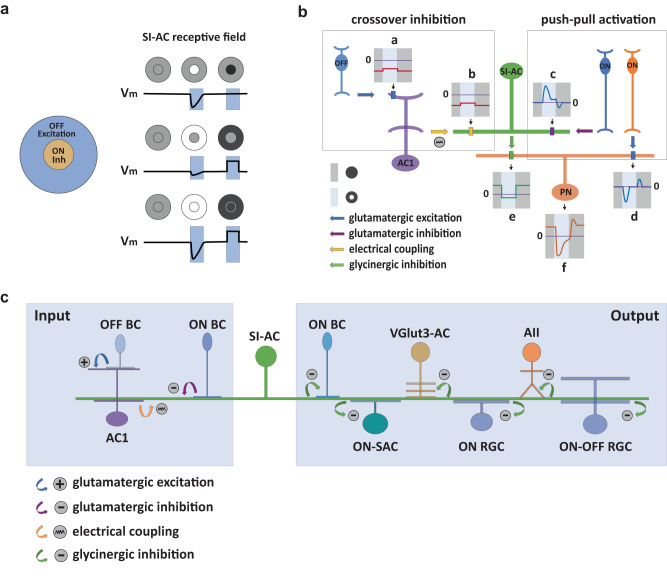
Unusual receptive field of SI-AC allows for crossover inhibition and push-pull activation. **a** SI-AC center-surround receptive field organization. SI-AC has a strong inhibitory “ON center” (top panel, middle) with a weak inhibitory “ON surround” (middle panel, middle), and an excitatory “OFF surround” (middle panel, right) without an opposed “OFF center” (top panel, right). Consequently, full-field ON stimulation produces an inhibition composed of both center and surround inhibitions (bottom panel, middle), while full-field OFF stimulation produces an excitation only from the surround (bottom panel, right). **b** Schematic diagrams of SI-AC mediated OFF to ON crossover inhibition. **a** OFF bipolar cells are activated by a dark background (OFF, dark gray bar), leading to a tonic inward current (red trace) in the wide-field AC1. The inward current is slightly reduced by a light spot in the center (ON, light blue bar). **b** AC1 transfers the inward current to SI-AC via an electrical synapse. **c** A light spot in the dark activates the ON bipolar cell (blue) which produces a large outward current at ON and a small inward current in OFF (blue trace) in the SI-AC via sign-inverting mGluR8 receptors. **d** The same (blue) or a different (orange) bipolar cell produces a large inward current at ON and a small outward current in OFF in neurons (PN) postsynaptic to the SI-AC. **e** Currents from AC1 (**b**) and ON bipolar cells (**c**) combine in the SI-AC to gate a Ca^2+^ conductance that leads to a tonic suppression in glycine release and an outward current during OFF and an inward current during ON in the postsynaptic neuron. **f** The currents from SI-AC (**e**) and ON bipolar cells (**d**) combine in the postsynaptic neuron, resulting in an enhanced inward current during ON together with a large outward current during OFF. **c** Model of SI-AC circuitry. In the dark, OFF bipolar cells depolarized SI-AC via a bistratified AC at electrical synapses. Glycine released by SI-AC inhibits ACs (AIIs, VGlut3-ACs), ON-RGCs, and ON-OFF RGCs. A spot of light hyperpolarizes SI-AC via mGluR8, relieving the inhibition and increasing the excitability of postsynaptic neurons that respond during light.

ACs and RGCs usually have center-surround receptive fields, with an excitatory center produced by bipolar cell inputs and an inhibitory surround produced by AC inputs. The center and surround regions behave antagonistically to each other insofar as both center and surround are mediated by the same polarity, ON or OFF, bipolar cells. SI-AC receives a central inhibitory input from ON bipolar cells and a surrounding excitatory input from OFF bipolar cells mediated by an electrically coupled AC. This arrangenment creates an unique receptive field organization in SI-AC (Fig. 8a). First, it has an inhibitory “ON center” (Fig. 8a, top panel, middle), instead of an excitatory center. Second, it has an excitatory “OFF surround” (Fig. 8a, middle panel, right) instead of an inhibitory surround. The “OFF surround” has no antagonistic “OFF center” (Fig. 6b, c, Fig. 8a, top panel, right). The “ON surround” by GABAergic input (Fig. 8a, middle panel, middle) is weak (Supplementary Fig. 2b), and has the same sign (inhibitory) as the “ON center”. Altogether, the receptive field of SI-AC can be summarized as “inhibitory ON center–excitatory OFF surround”, both of which can contribute to SI-AC depolarization in the dark and hyperpolarization in the light.

### “Crossover inhibition” and “Push-pull activation” mediated by SI-AC

SI-AC is a glycinergic interneuron that has an inhibitory effect on postsynaptic neurons. The unique receptive field organization allows it to mediate crossover inhibition (Fig. 8b) with monostratified dendrites instead of the bistratified dendrites that are required for crossover inhibition via other ACs. In the dark, a large inward current flows into SI-AC (Fig. 8b, b) from OFF bipolar cells (Fig. 8b, a) via the wide-field AC (AC1). When a spot of light is applied in the receptive field center, SI-AC ON bipolar cells through mGluR8 receptors promote a large outward current (Fig. 8b, c). After combing the currents from AC1 (Fig. 8b, b) and the ON bipolar cells (Fig. 8b, c), SI-AC uses glycinergic transmission to produce an outward current in the dark and an inward current in the light (Fig. 8b, e) in postsynaptic RGCs, ACs, and bipolar cells. The current from SI-AC (Fig. 8b, e) then combines with the current from the ON bipolar cells (Fig. 8b, d) in the postsynaptic neuron, and produces an enhanced inward current in the light and an outward current in the dark (Fig. 8b, f). In this way, SI-AC controls the responses of the postsynaptic neurons with two coordinated mechanisms. First, it provides an OFF to ON crossover inhibition (Fig. 8b, a, b, e) to suppress the spontaneous activity of the postsynaptic neurons in the dark. Second, the mGluR8-mediated inhibition serves as a switch to relieve the crossover inhibition in the light. The combination of light-evoked local excitation from ON bipolar cells (Fig. 8b, d) and local relief of crossover inhibition from SI-AC (Fig. 8b, c, e) produces a push-pull activation to enhance the ON responses of postsynaptic neurons (Fig. 8b, f). The crossover inhibition and push-pull activation work together to increase the signal-to-noise ratio of ON responses and enhance light detection of postsynaptic neurons in the dark. We summarized the SI-AC circuitry in Fig. 8c.

## Discussion

We demonstrate a postsynaptic role for the group III receptor mGluR8 in mediating a sign-inverting response between ON bipolar cells and the SI-AC. With the exception of the ON bipolar cell receptor mGluR6, group III mGluRs have been shown to function predominately in the pre-synaptic terminal to modulate neurotransmitter release, either as auto-receptors in glutamatergic terminals, or as hetero-receptors in nearby GABAergic terminals. Although expressed at lower levels than mGluR4 and mGluR7, mGluR8 is widely distributed in the CNS including the cerebellum, olfactory bulb, hippocampus, and cortex^65–67^. At these sites, mGluR8 regulates exocytosis by affecting Ca^2+^ entry or by direct modulation of the release machinery^68–70^. In the retina, a group III receptor also modulates vesicle replenishment in cones^71^. Our results clearly show that transmission at the ON bipolar to SI-AC synapse relies on postsynaptic mGluR8. An important question is whether or not similar mechanisms are also utilized by mGluR8 or other group III mGluRs in the brain. Interestingly, mGluR4 has been shown to be expressed postynaptically in certain areas such as the hippocampus^72^. Recent studies have suggested roles for group III mGluRs in neurological and psychiatric disorders including Parkinson’s disease, chronic pain, drug abuse, seizure, depression, and anxiety^65,73^. Hence, we expect that additional examples of postsynaptic responses mediated by group III mGluRs, including mGluR8, will emerge.

Our current understanding of ON- and OFF-center light responses in RGCs is predicated on a fundamental dichotomy that was first established almost 50 years ago^74–76^. This organizational scheme explains the light-ON and -OFF responses in bistratified RGCs such as the ON-OFF direction-selective cell with separate dendritic trees that ramify both in sublamina a and b^77^, and is recently supported by the measured response polarities of almost 40 types of anatomically characterized RGCs^78^. In a minor exception to the rule, dopaminergic ACs^79^ and M1-type intrinsically photosensitive RGCs^80^, which also receive inputs from rod and cone photoreceptors, ramify at the top of sublamina *a* and yet depolarize in response to light. However, both types receive excitatory inputs at ribbon synapses located en passant in ON bipolar cell axons, and thus the general polarity rule is conserved^17^. Additionally, some OFF bipolar cells in the cat label for the GABA synthetic enzyme GAD_65_ and the vesicular transporter VGAT, which is localized to dendrites, but a synaptic effect of GABA release in the IPL has not been established^81^. Here, we demonstrate a major exception to the fundamental organizational plan: SI-AC dendrites exclusively ramify in the ON sublamina of the IPL yet hyperpolarize in response to light. Our results raise the possibility of additional AC types that ramify in sublamina *b* that have either OFF-center responses or mixed ON- and OFF-center responses with different time courses, as well as the possibility of AC types that ramify in sublamina *a* that have ON-center or mixed ON- and OFF-responses.

Tonic glycinergic inputs from SI-AC are likely to hyperpolarize postsynaptic ON cells in the dark. This inhibition will be relieved by a small light spot. The combination of local excitation and local relief of shunting inhibition, a push-pull effect, may enhance the sensitivity to dim light stimuli for the ON responses of RGCs^82^. The shunting inhibition on its own may suppress spontaneous RGC firing, thereby improving the signal-to-noise ratio as well. SBEM analysis shows that SI-AC (H22 NAC^25^;) provides outputs to other ACs, including ON-SACs, VGlut3-ACs, and AII ACs (David Berson, personal communication, also see ref. ^46^). In ON-SACs, our results are consistent with the idea that SI-ACs can contribute to a slow, sustained, and widespread inhibition via Glyα4 receptors that control ON-SACs output gain and direction selectivity^46^. VGlut3-AC is a bistratified AC that has ON-dominant responses in its ON sublamina processes and OFF-dominant responses in its OFF sublamina processes^60,83^. This glutamatergic AC provides excitatory inputs to the looming detection of W3 and transient OFF α RGCs, as well as direction-selective ON DSGCs and ON-OFF DSGCs^31,38,84,85^. While spanning the sublamina *a/b* border, it is proposed that the ON-OFF segregation of VGlut3-AC dendrites allows the cell to participate in motion detection circuits without changing the ON-OFF response polarities of postsynaptic RGCs^60,83,85^. However, the origins of the ON-OFF segregation within the compact VGlut3-ACs remain unclear. SI-AC contacts the ON but not the OFF dendrites of VGluT3-ACs. It is expected to hyperpolarize the ON dendrites in the dark while depolarizing them in the light, thus contributing to the ON-OFF segregation of VGlut3-ACs dendrites. In AII cells, which provide an essential link in the rod pathway that subserves night vision, SI-AC may suppress spontaneous activity in the dark while allowing the depolarizing responses in rod bipolar cells during dim light stimuli to more easily excite AIIs. Interestingly, AII is a glycinergic AC that carries crossover inhibition from the ON channel to the OFF channel. The potential inhibition from SI-AC to AII implies an interaction between the two major crossover inhibition pathways (ON to OFF, OFF to ON). The actual roles of SI-ACs in visual processing may vary on a case-to-case basis and need to be examined with cell-type specific silencing, which could be optimally performed using an intersectional strategy to target single AC types.

Crossover inhibition is usually mediated by bistratified ACs that receive glutamatergic excitation from bipolar cells with the dendrites in one sublamina, while sending inhibition to bipolar cells, ACs, and RGCs with the dendrites in the other sublamina. In contrast, SI-AC can process both synaptic input and output in the same dendrites, presenting a new form of local computation of crossover inhibition. The circuit design for SI-AC offers several advantages: (1) the efficient use of dendritic structures contributes to better utilization of cellular resources; (2) eliminating OFF dendrites from SI-AC removes redundant inhibition to the OFF pathway; (3) the ON inhibition and OFF excitation work together to control the glycine release from SI-AC, so as to enhance the responses of postsynaptic cells in the ON pathway; and, (4) the surround excitation and center inhibition of SI-AC produce a global crossover inhibition that can be controlled (relieved) locally with a small spot of light (Fig. 2e, f, h). The ability to control the crossover inhibition with spots that are only a few tens of microns in diameter could potentially increase the spatial resolution of the ON channel output.

## Methods

### Animals

Adult mice (8–12 weeks old) of either sex maintained in C57BL/6 congenic background were used for experiments. All animal procedures were performed in accordance with the Guide for the Care and Use of Laboratory Animals as adopted and promulgated by the US National Institutes of Health. All procedures for testing and handling were approved by the Institutional Animal Care and Use Committee of Northwestern University. Animals were provided food and water ad libitum and were maintained on a regular 14-hr light /10-hr dark Light/Dark cycle. Animals were purchased from The Jackson Laboratory: VGAT-iCreER (C57BL/6N-Tg(Slc32a1-icre/ERT2)3Gloss/J, JAX 016582), VGAT-Cre (Slc32a1^tm2(cre)Lowl^/J, JAX 016962), CMV-Cre (B6.C-Tg(CMV-cre)1Cgn/J, JAX 006054), Scg2-tTA (B6.Cg-Tg(Scg2-tTA)1Jt/J, JAX 008284), Ai93 (B6;129 S6-*Igs7*^*tm93.1(tetO-GCaMP6f)Hze*^/J, JAX 024103), Ai85 (B6;129S-*Igs7*^tm85.1(tetO-gltI/GFP*)Hze^/J, JAX 026260), Rosa26-LSL-Cas9 (JAX 026175). To activate iCreER, Tamoxifen (75 mg/kg body weight) was injected intraperitoneally at postnatal 8-12 weeks for 2 weeks. Seven days after the last induction, mice were subjected to recording. All animals were randomly assigned into the experimental and control groups, while maintaining a balance between females (~130 animals) and males (~130 animals).

### Immunohistochemistry and imaging

Mice were euthanized by isoflurane inhalation followed by cervical dislocation, eyes were fixed with 4% paraformaldehyde, and retinas were dissected from the eyecup. After washing with a modified phosphate buffer (PB) containing 0.5% Triton X-100 and 0.1% NaN_3_, pH 7.4 6 times each for 30 min, retinas were blocked for 2 days in modified PB containing 3% donkey serum. Retinas were then incubated with primary antibody for 5 days at 4 °C. After wash, retinas were incubated with donkey secondary antibody for another 2 days at 4 °C. The primary antibodies used were as follows: chicken anti-GFP (1:1000, Abcam ab13970), goat anti-acetyltransferase (1:500, Millipore AB144P), rabbit anti-GlyT1 (1:200, antibodies-online, ABIN1841935), rabbit anti-RFP (1:500, Rockland 600-401-379). Secondary antibodies were conjugated to Alexa Fluor 488, Cy3 or Cy5: donkey anti-chicken (1:200, Jackson ImmunoResearch 703-545-155), donkey anti-rabbit (1:200, Jackson ImmunoResearch 711-165-152), donkey anti-goat (1:200, Jackson ImmunoResearch 703-175-147). For neurobiotin labeling, 30 mM Neurobiotin (Vector Labs, SP-1120) was added to the pipette solution, and was detected by using streptavidin (1:200, Vector Labs, SA-1300-1).

Images were acquired with ×63, ×25, or ×10 objectives in a Zeiss LSM-510 Meta confocal microscope and processed with LSM Image software, Image J, and Photoshop. Z-stack images were obtained at 0.25 mm intervals with ×63 objective. To measure the size of the dendritic field, a convex polygon was drawn connecting the outermost tips of the dendrites, and the area within this contour was measured. The diameter of the dendritic field was calculated from the measured area by assuming the dendritic field is circular. Soma diameter was calculated in the same way. The stratification levels were determined from the upper and lower boundaries of GFP-labeled arbors relative to the choline acetyltransferase (ChAT)-positive bands (60 and 27% of the IPL) in XZ plane. Data are presented as mean ± SD.

### In situ hybridization/RNAscope

In situ hybridization was performed using RNAScope Multiplex Fluorescent Reagent kit v2 from Advanced Cell Diagnostics (ACD). Briefly, retinas of adult mice (3 months old) were fixed in 4% PFA for 1 day, embedded in paraffin, and then sectioned at 5 μm thickness onto glass slides. Retina sections were then deparaffinized in xylene, rehydrated in ethanol, and processed for RNA in situ hybridization as per ACD’s instructions. The probe targeting 1277– 2219 of mGluR8 transcript (NM_008174.2) was purchased from ACD (catalog # 521491-C2 Mm-Grm8).

### CRISPR knockdown

We designed guide RNA using online guide design resources CRISPOR (crispor.tefor.net). To knockdown *GRM8*, we targeted exon2 which contains the starting codon of the *GRM8* gene. Two target sites with the highest predicted targeting score were chosen. DNA oligos were synthesized and cloned into a backbone that uses the human U6 promoter to express gRNA (PX458, Addgene plasmid 48138). Subsequently, gRNA expression cassette was PCR-amplified and subcloned into an AAV plasmid expressing nuclear-localized tdTomato (2xNLS-tdTomato) from smCBA promoter^86^. The 2xNLS-tdTomato sequence was cloned from Addgene plasmid 104054 and the smCBA promoter sequence was cloned from pTR-SB-smCBA-V2-empty plasmid (gift from Dr. William Hauswirth, University of Florida)

The oligo sequence for cloning gRNA are:

#1 forward (5′ to 3′) caccGTATGCGCATTCCATCCGCC;

#1 reverse (5′ to 3′) aaacGGCGGATGGAATGCGCATAC;

#2 forward (5′ to 3′) caccGCAGTCACTAACCTTCGTGC;

#2 reverse (5′ to 3′) aaacGCACGAAGGTTAGTGACTGC;

### Viral packaging and injection

AAVs were packaged and purified as previously described^87^. In brief, AAVs were produced with polyethylenimine (PEI) transfection of HEK293 cells in adherent cell culture with AAV cis, AAV trans, and adenovirus helper plasmid pAdΔF6. AAVs from cells pellets and media were collected 72 hrs post transfection and purified by iodixanol gradient ultracentrifugation. Viruses were concentrated and formulated in PBS.

AAV injections were performed on 5–6-week-old mice. For CRISPR knockdown of mGluR8, 1 µl of *AAV2 (YF4)-smCBA-gRNA-2xNLS-tdTomato* (1 × 10^13^ genome copies/ml) was injected into the eyes of VGAT-iCreER;Scg2-tTA;Ai93;Rosa26-LSL-Cas9 mice. GCaMP6 imaging was performed 5 weeks after injection.

### Two-photon GCaMP6f and iGluSnFr imaging

Mice were dark-adapted for at least 2 hours. Eyes were enucleated immediately after euthanization and the retinas were dissected from the eyecup under infrared illumination and mounted vitreal side up in the recording chamber. Tissue was continuously superfused with oxygenated (95% O_2_–5% CO_2_) Ames medium at room temperature and imaged with a two-photon microscope system (Thorlabs) equipped with a Mai Tai DeepSee ultrafast laser (Spectra-Physics) tuned to 940 nm. Label cells were visualized with a ×20 objective (XLUMPLFLN, 1.0 numerical aperture, Olympus). Visual stimuli are projected onto the photoreceptors using a digital projector system (TI LightCrafter 4500 with modified LEDs). To measure spatial tuning, spot stimuli with variable diameter were centered on the receptive field and presented with 1 Hz temporal square-wave modulations (100% Michelson contrast) with the averaged intensity of ~1500 rhodopsin isomerizations/cone/s. To separate GCaMP6f signal acquisition from light stimulation, the projector LEDs (350 and 470 nm) were electronically gated by a copy of the resonant scanner trigger signal at 8kHZ so that the GCaMP6f or iGluSnFr signal is acquired during a forward scan sweep while the image is projected onto the retina during the return (discarded or unmonitored) sweep. ThorImageLS software (Thorlabs, Inc.) was used for Imaging acquisition. Data were analyzed with HCImage (Hamamatsu Photonics), and fluorescence values were the averages across the dendritic fields. Pharmacological reagents: (S) 3,4 DCPG (0.5 µM; Tocris Bioscience), gallein (100 µM; Tocris Bioscience), BaCl_2_ (100 µM; Sigma Millipore), tertiapin-Q (500 nM, Alomone Labs), UBP310 (25 µM; Tocris Bioscience), GYKI 53655 (25 µM; Tocris Bioscience), strychnine (5 µM; Sigma Millipore), gabazine (20 µM; Sigma Millipore), MFA (25 µM; Sigma Millipore), Z-Cyclopentyl-AP4 (50 µM; Tocris Bioscience), AMN-082 (10 µM; Tocris Bioscience), and ADX 71743 (7.4 µM; Tocris Bioscience) were added to the bath solution.

### Electrophysiology

The external solution is oxygenated Ames medium as used in two-photon imaging. Whole-cell recordings were made with patch pipettes (tip resistance 5–7 MΩ). Membrane current or potential was amplified, digitized at 10–20 kHz (Axopatch 700B amplifier; Digidata 1440 A Digitizer), stored, and analyzed by using pClamp 10.0 (Molecular Devices). Pipettes were filled with an intracellular solution composed of (in mM): 125 K-gluconate, 10 NaCl, 1 MgCl_2__,_ 10 EGTA, 5 HEPES, 5-ATP-Na, 0.1 GTP-Na (280 mOsm; pH adjusted to 7.4 with KOH). For gallein, BaCl_2_, and tertiapin-Q experiments, 10 µM gallein, 10 µM BaCl_2,_ or 100 nM tertiapin-Q was added to the pipette solution. For the neurobiotin filling, 30 mM Neurobiotin was added to the pipette solution. Absolute voltage values were corrected for a liquid junction potential of −12.8 mV.

### Statistics

Mice of each sex were used in all experiments. OriginPro 2021 (OriginLab Corporation) was used for statistical analysis. Data are reported as mean ± SEM unless indicated otherwise. The box plot displays the mean, 25th, and 75th percentiles, while the whiskers indicate the 1.5 interquartile range. Statistical comparison was made based on Wilcoxon Signed Rank test or Mann–Whitney test, significance was accepted at *p* < 0.05.

### Reporting summary

Further information on research design is available in the Nature Portfolio Reporting Summary linked to this article.

### Supplementary information

Supplementary information

Reporting summary

### Source data

Source Data

## Data Availability

The data that support the findings of this study are available from the corresponding authors. The data generated in this study are available in the paper, supplementary information, and source data. Source data are provided with this paper.

## References

[1] Wassle H, Boycott BB (1991). Functional architecture of the mammalian retina. Physiol. Rev..

[2] Massey, S. In: Progress in retinal research, Vol. 9 (ed Chader G. Osborne N. N.) Ch. p 399–425, (Pergamon Press, 1990).

[3] Masland RH (2001). The fundamental plan of the retina. Nat. Neurosci..

[4] Werblin FS, Dowling JE (1969). Organization of the retina of the mudpuppy, Necturus maculosus. II. Intracellular recording. J. Neurophysiol..

[5] Kaneko A (1970). Physiological and morphological identification of horizontal, bipolar and amacrine cells in goldfish retina. J. Physiol..

[6] DeVries SH, Schwartz EA (1999). Kainate receptors mediate synaptic transmission between cones and ‘Off’ bipolar cells in a mammalian retina. Nature.

[7] Slaughter MM, Miller RF (1981). 2-amino-4-phosphonobutyric acid: a new pharmacological tool for retina research. Science.

[8] Masu M (1995). Specific deficit of the ON response in visual transmission by targeted disruption of the mGluR6 gene. Cell.

[9] Slaughter MM, Miller RF (1983). Bipolar cells in the mudpuppy retina use an excitatory amino acid neurotransmitter. Nature.

[10] Ehinger B, Ottersen OP, Storm-Mathisen J, Dowling JE (1988). Bipolar cells in the turtle retina are strongly immunoreactive for glutamate. Proc. Natl Acad. Sci. USA.

[11] Palmer MJ (2010). Characterisation of bipolar cell synaptic transmission in goldfish retina using paired recordings. J. Physiol..

[12] Euler T, Schneider H, Wassle H (1996). Glutamate responses of bipolar cells in a slice preparation of the rat retina. J. Neurosci..

[13] Baden T, Berens P, Bethge M, Euler T (2013). Spikes in mammalian bipolar cells support temporal layering of the inner retina. Curr. Biol..

[14] Franke K (2017). Inhibition decorrelates visual feature representations in the inner retina. Nature.

[15] Borghuis BG, Marvin JS, Looger LL, Demb JB (2013). Two-photon imaging of nonlinear glutamate release dynamics at bipolar cell synapses in the mouse retina. J. Neurosci..

[16] Singer JH, Diamond JS (2003). Sustained Ca2+ entry elicits transient postsynaptic currents at a retinal ribbon synapse. J. Neurosci..

[17] Dumitrescu ON, Pucci FG, Wong KY, Berson DM (2009). Ectopic retinal ON bipolar cell synapses in the OFF inner plexiform layer: contacts with dopaminergic amacrine cells and melanopsin ganglion cells. J. Comp. Neurol..

[18] Molnar A, Hsueh HA, Roska B, Werblin FS (2009). Crossover inhibition in the retina: circuitry that compensates for nonlinear rectifying synaptic transmission. J. Comput. Neurosci..

[19] Werblin FS (2010). Six different roles for crossover inhibition in the retina: correcting the nonlinearities of synaptic transmission. Vis. Neurosci..

[20] Manookin MB, Beaudoin DL, Ernst ZR, Flagel LJ, Demb JB (2008). Disinhibition combines with excitation to extend the operating range of the OFF visual pathway in daylight. J. Neurosci..

[21] Rosa JM, Ruehle S, Ding H, Lagnado L (2016). Crossover inhibition generates sustained visual responses in the inner retina. Neuron.

[22] Peng YR (2019). Molecular classification and comparative taxonomics of foveal and peripheral cells in primate retina. Cell.

[23] Yan W (2020). Mouse retinal cell atlas: molecular identification of over sixty amacrine cell types. J. Neurosci..

[24] Badea TC, Nathans J (2004). Quantitative analysis of neuronal morphologies in the mouse retina visualized by using a genetically directed reporter. J. Comp. Neurol..

[25] Helmstaedter M (2013). Connectomic reconstruction of the inner plexiform layer in the mouse retina. Nature.

[26] MacNeil MA, Masland RH (1998). Extreme diversity among amacrine cells: implications for function. Neuron.

[27] Vaney DI, Sivyer B, Taylor WR (2012). Direction selectivity in the retina: symmetry and asymmetry in structure and function. Nat. Rev. Neurosci..

[28] Euler T, Detwiler PB, Denk W (2002). Directionally selective calcium signals in dendrites of starburst amacrine cells. Nature.

[29] Grimes WN, Zhang J, Graydon CW, Kachar B, Diamond JS (2010). Retinal parallel processors: more than 100 independent microcircuits operate within a single interneuron. Neuron.

[30] Lee S, Zhang Y, Chen M, Zhou ZJ (2016). Segregated glycine-glutamate co-transmission from vGluT3 amacrine cells to contrast-suppressed and contrast-enhanced retinal circuits. Neuron.

[31] Kim, T., Soto, F. & Kerschensteiner, D. An excitatory amacrine cell detects object motion and provides feature-selective input to ganglion cells in the mouse retina. *Elife* **4**10.7554/eLife.08025 (2015). 10.7554/eLife.08025 PMC4467229 25988808

[32] Park SJH (2018). Convergence and divergence of CRH amacrine cells in mouse retinal circuitry. J. Neurosci..

[33] Jacoby J, Zhu Y, DeVries SH, Schwartz GW (2015). An amacrine cell circuit for signaling steady illumination in the retina. Cell Rep..

[34] Park SJ (2015). Function and circuitry of VIP+ interneurons in the mouse retina. J. Neurosci..

[35] Kerstein PC, Leffler J, Sivyer B, Taylor WR, Wright KM (2020). Gbx2 identifies two amacrine cell subtypes with distinct molecular, morphological, and physiological properties. Cell Rep..

[36] Grimes, W. N. et al. A high-density narrow-field inhibitory retinal interneuron with direct coupling to Muller glia. *J. Neurosci.*10.1523/JNEUROSCI.0199-20.2021 (2021). 10.1523/JNEUROSCI.0199-20.2021 PMC8276741 34083252

[37] Poleg-Polsky A, Ding H, Diamond JS (2018). Functional compartmentalization within starburst amacrine cell dendrites in the retina. Cell Rep..

[38] Lee S (2014). An unconventional glutamatergic circuit in the retina formed by vGluT3 amacrine cells. Neuron.

[39] Demb JB, Singer JH (2012). Intrinsic properties and functional circuitry of the AII amacrine cell. Vis. Neurosci..

[40] Kim T, Kerschensteiner D (2017). Inhibitory control of feature selectivity in an object motion sensitive circuit of the retina. Cell Rep..

[41] Daigle TL (2018). A suite of transgenic driver and reporter mouse lines with enhanced brain-cell-type targeting and functionality. Cell.

[42] Madisen L (2015). Transgenic mice for intersectional targeting of neural sensors and effectors with high specificity and performance. Neuron.

[43] Gossen M, Bujard H (1992). Tight control of gene expression in mammalian cells by tetracycline-responsive promoters. Proc. Natl. Acad. Sci. USA.

[44] Hong HK (2007). Inducible and reversible Clock gene expression in brain using the tTA system for the study of circadian behavior. PLoS Genet..

[45] Kay JN, Voinescu PE, Chu MW, Sanes JR (2011). Neurod6 expression defines new retinal amacrine cell subtypes and regulates their fate. Nat. Neurosci..

[46] Jain V (2022). Gain control by sparse, ultra-slow glycinergic synapses. Cell Rep..

[47] Conn PJ, Pin JP (1997). Pharmacology and functions of metabotropic glutamate receptors. Annu. Rev. Pharm. Toxicol..

[48] Thomas, N. K. et al. S)-3,4-DCPG, a potent and selective mGlu8a receptor agonist, activates metabotropic glutamate receptors on primary afferent terminals in the neonatal rat spinal cord. *Neuropharmacology* **40**, 311–318 (2001). 10.1016/s0028-3908(00)00169-6 11166323

[49] Quraishi S, Reed BT, Duvoisin RM, Taylor WR (2010). Selective activation of mGluR8 receptors modulates retinal ganglion cell light responses. Neuroscience.

[50] Jones PJ, Xiang Z, Conn PJ (2008). Metabotropic glutamate receptors mGluR4 and mGluR8 regulate transmission in the lateral olfactory tract-piriform cortex synapse. Neuropharmacology.

[51] Mitsukawa K (2005). A selective metabotropic glutamate receptor 7 agonist: activation of receptor signaling via an allosteric site modulates stress parameters in vivo. Proc. Natl. Acad. Sci. USA.

[52] Kalinichev M (2013). ADX71743, a potent and selective negative allosteric modulator of metabotropic glutamate receptor 7: in vitro and in vivo characterization. J. Pharm. Exp. Ther..

[53] Zhong L (2008). Next generation of adeno-associated virus 2 vectors: point mutations in tyrosines lead to high-efficiency transduction at lower doses. Proc. Natl. Acad. Sci. USA.

[54] Petrs-Silva H (2011). Novel properties of tyrosine-mutant AAV2 vectors in the mouse retina. Mol. Ther..

[55] Medina I (2000). A switch mechanism for G beta gamma activation of I(KACh). J. Biol. Chem..

[56] Dascal N, Kahanovitch U (2015). The roles of gbetagamma and galpha in gating and regulation of GIRK channels. Int. Rev. Neurobiol..

[57] Cui Y (2018). Astroglial Kir4.1 in the lateral habenula drives neuronal bursts in depression. Nature.

[58] Djukic B, Casper KB, Philpot BD, Chin LS, McCarthy KD (2007). Conditional knock-out of Kir4.1 leads to glial membrane depolarization, inhibition of potassium and glutamate uptake, and enhanced short-term synaptic potentiation. J. Neurosci..

[59] Chen X, Johnston D (2005). Constitutively active G-protein-gated inwardly rectifying K+ channels in dendrites of hippocampal CA1 pyramidal neurons. J. Neurosci..

[60] Chen M, Lee S, Zhou ZJ (2017). Local synaptic integration enables ON-OFF asymmetric and layer-specific visual information processing in vGluT3 amacrine cell dendrites. Proc. Natl. Acad. Sci. USA.

[61] Jin W, Lu Z (1999). Synthesis of a stable form of tertiapin: a high-affinity inhibitor for inward-rectifier K+ channels. Biochemistry.

[62] Huang Y (2018). GIRK1-mediated inwardly rectifying potassium current suppresses the epileptiform burst activities and the potential antiepileptic effect of ML297. Biomed. Pharmacother..

[63] Zhou JY, Siderovski DP, Miller RJ (2000). Selective regulation of N-type Ca channels by different combinations of G-protein beta/gamma subunits and RGS proteins. J. Neurosci..

[64] Herlitze S (1996). Modulation of Ca2+ channels by G-protein beta gamma subunits. Nature.

[65] Niswender CM, Conn PJ (2010). Metabotropic glutamate receptors: physiology, pharmacology, and disease. Annu. Rev. Pharm. Toxicol..

[66] Ferraguti F, Shigemoto R (2006). Metabotropic glutamate receptors. Cell Tissue Res..

[67] Duvoisin RM, Zhang C, Ramonell K (1995). A novel metabotropic glutamate receptor expressed in the retina and olfactory bulb. J. Neurosci..

[68] Cartmell J, Schoepp DD (2000). Regulation of neurotransmitter release by metabotropic glutamate receptors. J. Neurochem..

[69] Mercier MS, Lodge D (2014). Group III metabotropic glutamate receptors: pharmacology, physiology and therapeutic potential. Neurochem. Res..

[70] Duvoisin RM (2010). Acute pharmacological modulation of mGluR8 reduces measures of anxiety. Behav. Brain Res..

[71] Van Hook MJ (2017). A presynaptic group III mGluR recruits Gbetagamma/SNARE interactions to inhibit synaptic transmission by cone photoreceptors in the vertebrate retina. J. Neurosci..

[72] Bradley SR, Levey AI, Hersch SM, Conn PJ (1996). Immunocytochemical localization of group III metabotropic glutamate receptors in the hippocampus with subtype-specific antibodies. J. Neurosci..

[73] Crupi R, Impellizzeri D, Cuzzocrea S (2019). Role of metabotropic glutamate receptors in neurological disorders. Front. Mol. Neurosci..

[74] Famiglietti EV, Kaneko A, Tachibana M (1977). Neuronal architecture of on and off pathways to ganglion cells in carp retina. Science.

[75] Famiglietti EV, Kolb H (1976). Structural basis for ON-and OFF-center responses in retinal ganglion cells. Science.

[76] Nelson R, Famiglietti EV, Kolb H (1978). Intracellular staining reveals different levels of stratification for on- and off-center ganglion cells in cat retina. J. Neurophysiol..

[77] Bloomfield SA, Miller RF (1986). A functional organization of ON and OFF pathways in the rabbit retina. J. Neurosci..

[78] Bae JA (2018). Digital museum of retinal ganglion cells with dense anatomy and physiology. Cell.

[79] Zhang DQ, Zhou TR, McMahon DG (2007). Functional heterogeneity of retinal dopaminergic neurons underlying their multiple roles in vision. J. Neurosci..

[80] Wong KY, Dunn FA, Graham DM, Berson DM (2007). Synaptic influences on rat ganglion-cell photoreceptors. J. Physiol..

[81] Kao YH (2004). Evidence that certain retinal bipolar cells use both glutamate and GABA. J. Comp. Neurol..

[82] Euler T, Masland RH (2000). Light-evoked responses of bipolar cells in a mammalian retina. J. Neurophysiol..

[83] Hsiang, J. C., Johnson, K. P., Madisen, L., Zeng, H. & Kerschensteiner, D. Local processing in neurites of VGluT3-expressing amacrine cells differentially organizes visual information. *Elife* **6**10.7554/eLife.31307 (2017). 10.7554/eLife.31307 PMC5653236 29022876

[84] Krishnaswamy A, Yamagata M, Duan X, Hong YK, Sanes JR (2015). Sidekick 2 directs formation of a retinal circuit that detects differential motion. Nature.

[85] Kim, T., Shen, N., Hsiang, J. C., Johnson, K. P. & Kerschensteiner, D. Dendritic and parallel processing of visual threats in the retina control defensive responses. *Sci. Adv.* **6**10.1126/sciadv.abc9920 (2020). 10.1126/sciadv.abc9920 PMC7673819 33208370

[86] Haire SE (2006). Light-driven cone arrestin translocation in cones of postnatal guanylate cyclase-1 knockout mouse retina treated with AAV-GC1. Invest. Ophthalmol. Vis. Sci..

[87] Xu J (2022). Intersectional mapping of multi-transmitter neurons and other cell types in the brain. Cell Rep..

