# Supplementary material for: A sign-inverted receptive field of inhibitory interneurons provides a pathway for ON-OFF interactions in the retina
Source: Nat Commun. 2023 Sep 23;14:5937. doi: 10.1038/s41467-023-41638-3 (PMC10517963; doi:10.1038/s41467-023-41638-3)
Supplement: Supplementary file 1 — Supplementary information [file 41467_2023_41638_MOESM1_ESM.pdf]

Supplementary information

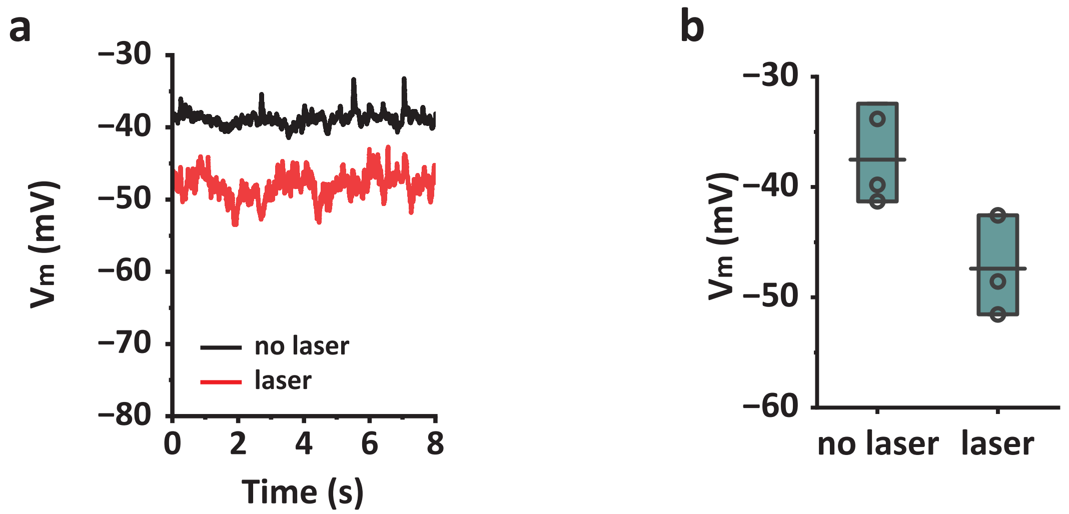

Supplementary Figure 1. Estimation of direct two-photon laser excitation of the photoreceptors. a. Whole-cell recording of membrane potential of a SI-AC with or without two-photon laser excitation. It is important to consider the photoactivating effect of unfocused infrared laser light on photoreceptor outer segments when comparing the results from GCaMP6 imaging and whole-cell recording. To measure this photoactivation, we recorded the Vm of SI-AC before and during laser scanning and observed a sustained hyperpolarization. By comparing the hyperpolarizing effect of two-photon laser illumination during current clamp recording (~9.8mV) with the effect of applying a background light in the absence of scanning (Figure 2j, orange curve), we conclude that the two-photon laser produces a hyperpolarizing response equivalent to ~1000 R*/rod/s. As a result, the total light exposure was 2500 R*/rod/s, while the dark condition maintained an intensity of 1000 R*/rod/s. **b.** Summary of membrane potential with or without two-photon laser excitation. N=3 cells for both. The box plot displays the mean, 25th, and 75th percentiles, while the whiskers indicate the 1.5 interquartile range. Source data are provided as a Source Data file. Experiments were performed on VGAT-iCreER;Scg2-tTA;Ai93 mice.

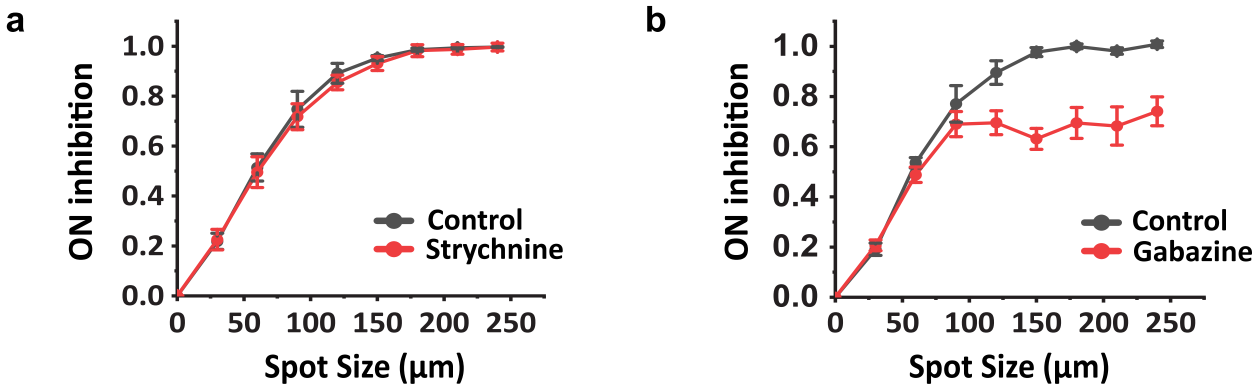

Supplementary Figure 2. Contributions of GABAergic and glycinergic inhibition to ON inhibition/hyperpolarization of SI-AC. a. 10µM Strychnine (glycine receptor antagonist) had no effect on the ON inhibition, n=9 cells. Error Bars: SEM. ns: *p* = 0.055, Wilcoxon Signed Rank test, two tailed. **b.** 50µM Gabazine (GABAa antagonist) decreased the ON inhibition at spots larger than 100µm, n=5 cells. Error Bars: SEM. **p* = 0.016, Wilcoxon Signed Rank test, two tailed. Experiments were performed on VGAT-iCreER;Scg2-tTA;Ai93 mice.

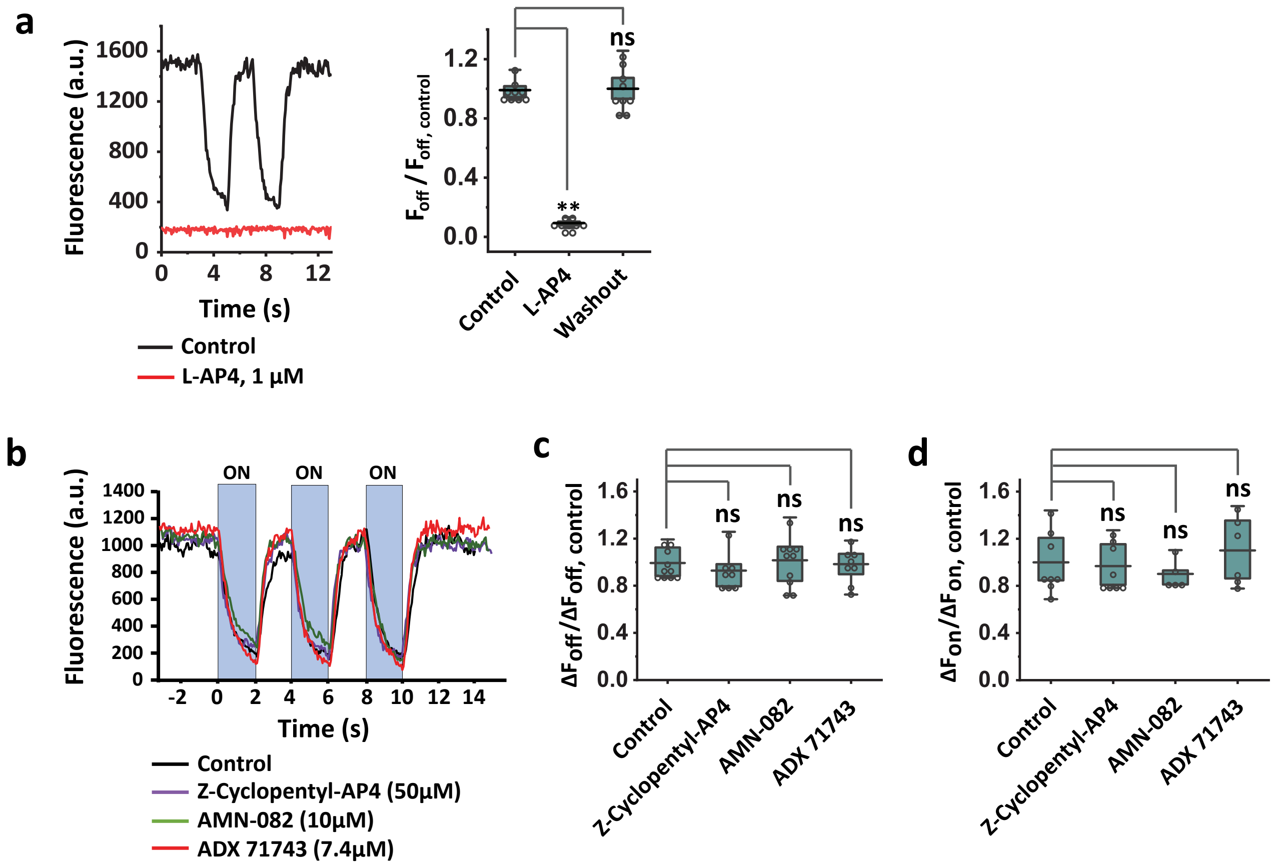

Supplementary Figure 3. mGluR4 and mGluR7 didn't mediate ON inhibition. a L-AP4 (1µM), the group III mGluRs agonist, abolished GCaMP responses in the dark and light, n=9 cells. $^{**}p = 0.002$, Wilcoxon Signed Rank test, one tailed. ns: $p = 0.91$, Wilcoxon Signed Rank test, two tailed. **b-d** Z-Cyclopentyl-AP4 (group III mGluR agonist more potent on mGluR4 than mGluR8), AMN-082 (selective agonist for mGluR7) and ADX 71743 (a negative allosteric modulator for mGluR7) had no effect on the responses (**d**) or resting signal (**c**) in SI-AC. **c** n=10 cells for control, n= 8 cells for Z-Cyclopentyl-AP4 (ns: $p = 0.38$), n=10 cells for AMN-082 (ns: $p = 1.0$), n= 8 cells for ADX 71743 (ns: $p = 0.94$), Wilcoxon Signed Rank test, two tailed. **d** n=8 cells for control, n=8 cells for Z-Cyclopentyl-AP4 (ns: $p = 0.84$), n=5 cells for AMN-082 (ns: $p = 0.13$), n= 6 cells for ADX 71743 (ns: $p = 0.84$), Wilcoxon Signed Rank test, two tailed. The box plots display the mean, 25th, and 75th percentiles, while the whiskers indicate the 1.5 interquartile range. Source data are provided as a Source Data file. Experiments were performed on VGAT-iCreER;Scg2-tTA;Ai93 mice.

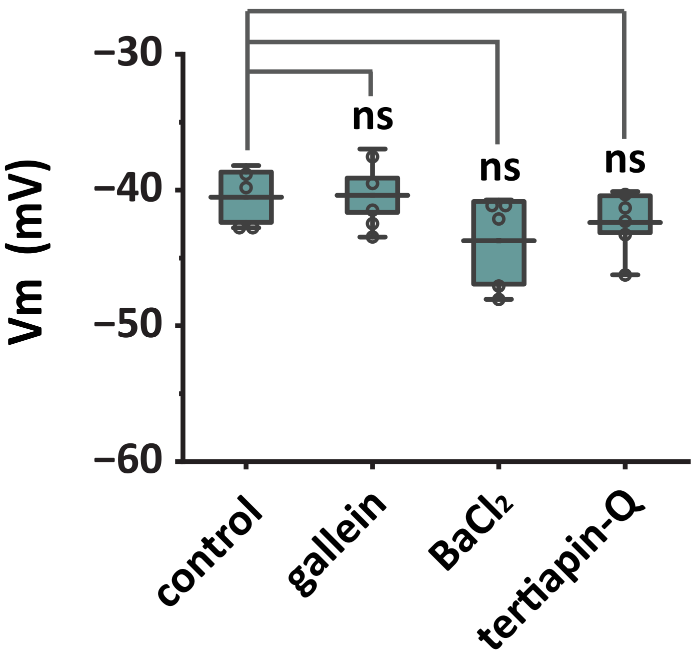

Supplementary Figure 4. Gallein, $BaCl_2$, and tertiapin-Q had no effect on membrane potential in the dark. N=5 cells for all. ns: p = 0.67 (gallein), p = 0.40 ($BaCl_2$), p = 0.27 (tertiapin-Q), Mann-Whitney Test, two tailed. The box plot displays the mean, 25th, and 75th percentiles, while the whiskers indicate the 1.5 interquartile range. Source data are provided as a Source Data file. Experiments were performed in VGAT-iCreER;Scg2-tTA;Ai93 mice.

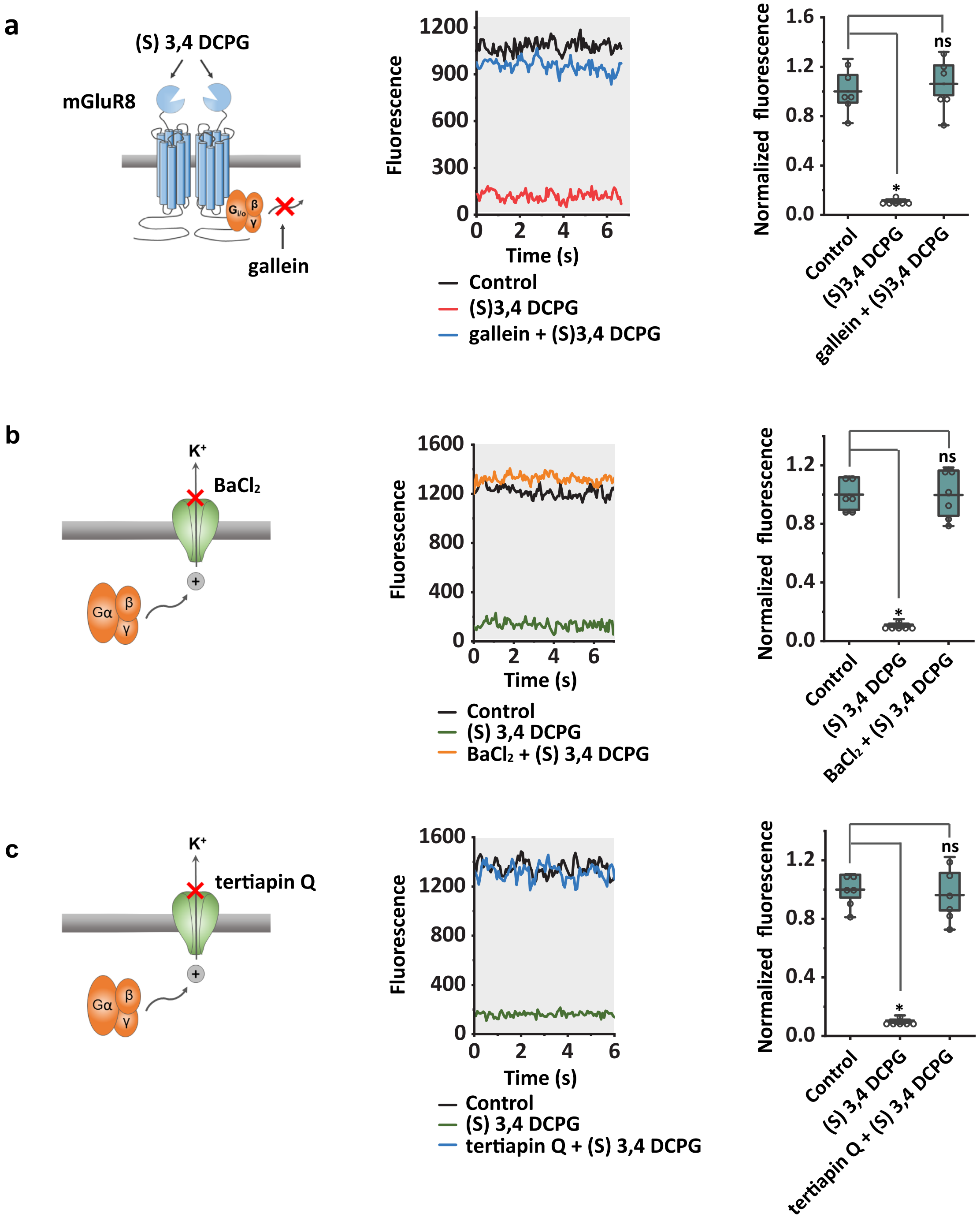

Supplementary Figure 5. Decrease in the intracellular Ca^{2+} concentration in ON inhibition is exclusively mediated by G-protein βγ subunits and GIRK channels. In this scenario, the mGluR8 agonist (S) 3,4 DCPG (0.5μM) acts directly on SI-AC to and reduces the intracellular Ca^{2+} concentration. Gallein (100 μM) (**a**), $BaCl_2$ (100 μM) (**b**), and tertiapin-Q (500 nM) (**c**) in the bath completely reverse the effect of (S) 3,4 DCPG (0.5μM) on the GCaMP6 signal (n=6 cells for all), suggesting the decrease in the intracellular Ca^{2+} concentration in ON inhibition was exclusively mediated by G-protein βγ subunits and GIRK channels. **a** *p = 0.031, ns: p = 1.00. **b** *p = 0.031, ns: p = 0.68. **c** *p = 0.031, ns: p = 0.69. Wilcoxon Signed Rank test, two tailed. The box plots display the mean, 25th, and 75th percentiles, while the whiskers indicate the 1.5 interquartile range. Source data are provided as a Source Data file. Experiments were performed in VGAT-iCreER;Scg2-tTA;Ai93 mice.

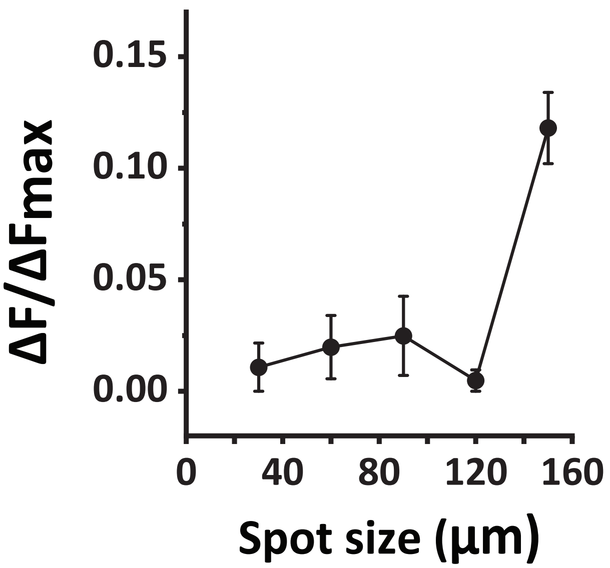

Supplementary Figure 6. Spatial profile of OFF responses to small dark spots. The OFF responses were measured with a dark spot (0% contrast) with enlarging diameter presented on a gray background (50% contrast). $\Delta F/\Delta F_{max} = (F - F_{0\mu m})/(F_{900\mu m} - F_{0\mu m})$, as in Figure 7c. N=5 cells. Error bars: SEM. Experiments were performed in VGAT-iCreER;Scg2-tTA;Ai93 mice.

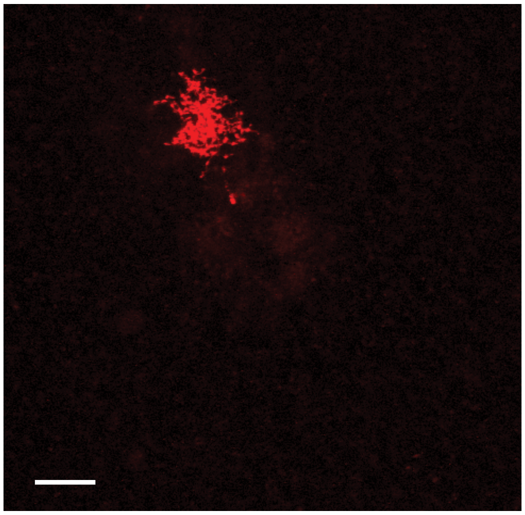

Supplementary Figure 7. MFA in the bath abolished labeling of the wide-field AC. Scale bar: 25μm. Experiments were replicated independently in at least 8 cells with similar results.

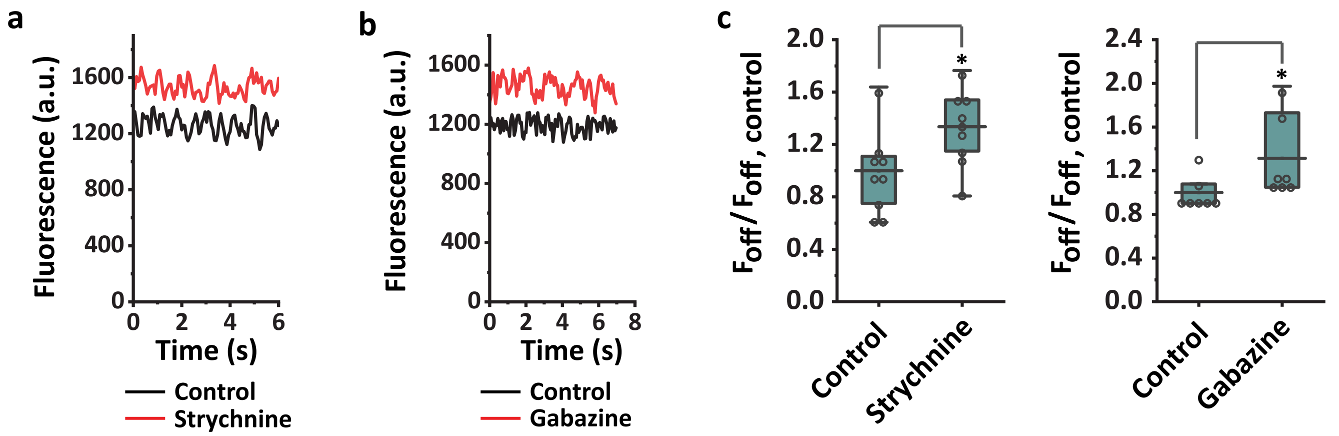

Supplementary Figure 8. SI-AC received a small, tonic level of GABAergic and Glycinergic inhibition from other ACs in the dark. a-b Strychnine (**a**) and Gabazine (**b**) slightly enhanced GCaMP6 signals in the dark. **c** Summary of the effects of Strychnine and Gabazine on GCaMP6 OFF response, n=9 cells for Strychnine (*p = 0.039), n=7 cells for Gabazine (*p = 0.031). Wilcoxon Signed Rank test, two tailed. The box plots display the mean, 25th, and 75th percentiles, while the whiskers indicate the 1.5 interquartile range. Source data are provided as a Source Data file. Experiments were performed in VGAT-iCreER;Scg2-tTA;Ai93 mice.
