# Supplementary material for: A sign-inverted receptive field of inhibitory interneurons provides a pathway for ON-OFF interactions in the retina
Source: Nat Commun. 2023 Sep 23;14:5937. doi: 10.1038/s41467-023-41638-3 (PMC10517963; doi:10.1038/s41467-023-41638-3)
Supplement: Supplementary file 2 — Reporting summary [file 41467_2023_41638_MOESM2_ESM.pdf]

nature portfolio

Corresponding author(s): Yongling Zhu, Steven H. DeVries
Last updated by author(s): 2023-7-30

Reporting Summary

Nature Portfolio wishes to improve the reproducibility of the work that we publish. This form provides structure for consistency and transparency in reporting. For further information on Nature Portfolio policies, see our Editorial Policies and the Editorial Policy Checklist.

Please do not complete any field with "not applicable" or n/a. Refer to the help text for what text to use if an item is not relevant to your study.
For final submission: please carefully check your responses for accuracy; you will not be able to make changes later.

Statistics

For all statistical analyses, confirm that the following items are present in the figure legend, table legend, main text, or Methods section.

n/a	Confirmed	
☐	☒	The exact sample size (*n*) for each experimental group/condition, given as a discrete number and unit of measurement
☐	☒	A statement on whether measurements were taken from distinct samples or whether the same sample was measured repeatedly
☐	☒	The statistical test(s) used AND whether they are one- or two-sided<br>*Only common tests should be described solely by name; describe more complex techniques in the Methods section.*
☒	☐	A description of all covariates tested
☒	☐	A description of any assumptions or corrections, such as tests of normality and adjustment for multiple comparisons
☐	☒	A full description of the statistical parameters including central tendency (e.g. means) or other basic estimates (e.g. regression coefficient) AND variation (e.g. standard deviation) or associated estimates of uncertainty (e.g. confidence intervals)
☐	☒	For null hypothesis testing, the test statistic (e.g. *F*, *t*, *r*) with confidence intervals, effect sizes, degrees of freedom and *P* value noted<br>*Give P values as exact values whenever suitable.*
☒	☐	For Bayesian analysis, information on the choice of priors and Markov chain Monte Carlo settings
☒	☐	For hierarchical and complex designs, identification of the appropriate level for tests and full reporting of outcomes
☒	☐	Estimates of effect sizes (e.g. Cohen's *d*, Pearson's *r*), indicating how they were calculated

Our web collection on statistics for biologists contains articles on many of the points above.

Software and code

Policy information about availability of computer code

Data collection	ThorImageLS software (Thorlabs, Inc) was used for Imaging acquisition. pCLAMP 10 (Molecular Devices) was used for electrophysiology acquisition.
Data analysis	HCImage (Hamamatsu Photonics) was used for imaging analysis. pCLAMP 10 (Molecular Devices) was used for electrophysiology analysis. OriginPro 2021(OriginLab Corporation) was used for statistical analysis.

For manuscripts utilizing custom algorithms or software that are central to the research but not yet described in published literature, software must be made available to editors and reviewers. We strongly encourage code deposition in a community repository (e.g. GitHub). See the Nature Portfolio guidelines for submitting code & software for further information.

Data

Policy information about availability of data

All manuscripts must include a data availability statement. This statement should provide the following information, where applicable:
- Accession codes, unique identifiers, or web links for publicly available datasets
- A description of any restrictions on data availability
- For clinical datasets or third party data, please ensure that the statement adheres to our policy

The data that support the findings of this study are available from the corresponding authors. Source data are provided with this paper. All data supporting the findings are provided in the paper, supplementary information, and source data.

Research involving human participants, their data, or biological material

Policy information about studies with human participants or human data. See also policy information about sex, gender (identity/presentation), and sexual orientation and race, ethnicity and racism.

Reporting on sex and gender	NA
Reporting on race, ethnicity, or other socially relevant groupings	NA
Population characteristics	NA
Recruitment	NA
Ethics oversight	NA

Note that full information on the approval of the study protocol must also be provided in the manuscript.

Field-specific reporting

Please select the one below that is the best fit for your research. If you are not sure, read the appropriate sections before making your selection.

☒ Life sciences ☐ Behavioural & social sciences ☐ Ecological, evolutionary & environmental sciences

For a reference copy of the document with all sections, see nature.com/documents/nr-reporting-summary-flat.pdf

Life sciences study design

All studies must disclose on these points even when the disclosure is negative.

Sample size	Our sample size was determined by running a power analysis based on the effect-size estimate observed in previous similar studies (e.g., Grabner et al, 2016).
Data exclusions	Imaging data was only excluded from animals where GCaMP6 expression was unacceptably low due to failed Tamoxifen induction.
Replication	Calcium imaging and whole-cell recording experiments were independently replicated in a minimum of 5 cells. Each replication attempt yielded successful results, and all recorded cells are represented in the data. Immunohistochemistry experiments were also independently replicated in at least 15 cells, showing similar results. Neurobiotin tracing experiments were independently replicated in a minimum of 6 cells.
Randomization	All animals were randomly assigned into the experimental and control groups, while maintaining a balance between females and males.
Blinding	The investigators could not be blinded to pharmacological experiments because they were required to administer the drug, perform recording, and conduct data analysis.

Reporting for specific materials, systems and methods

We require information from authors about some types of materials, experimental systems and methods used in many studies. Here, indicate whether each material, system or method listed is relevant to your study. If you are not sure if a list item applies to your research, read the appropriate section before selecting a response.

Materials & experimental systems

n/a	Involved in the study	
☐	☒	Antibodies
☐	☒	Eukaryotic cell lines
☒	☐	Palaeontology and archaeology
☐	☒	Animals and other organisms
☒	☐	Clinical data
☒	☐	Dual use research of concern
☒	☐	Plants

Methods

n/a	Involved in the study	
☒	☐	ChIP-seq
☒	☐	Flow cytometry
☒	☐	MRI-based neuroimaging

Antibodies

Antibodies used	Chicken anti-GFP (1:1000, Abcam ab13970),<br>Goat anti-acetyltransferase (1:500, Millipore AB144P)<br>Rabbit anti-GlyT1 (1:200, antibodies-online, ABIN1841935)<br>Rabbit anti-RFP (1:500, Rockland 600-401-379)<br>Donkey anti-chicken (1:200, JacksonImmuno #703-545-155)<br>Donkey anti-rabbit (1:200, JacksonImmuno, #711-165-152)<br>Donkey anti-goat (1:200, JacksonImmuno, #703-175-147)
Validation	The antibodies have been validated extensively. For anti-GFP, please see references PMID: 34463618, PMID: 33755020; For anti-acetyltransferase, please see references PMID: 26052670, PMID: 25868900; For anti-GlyT1, please see reference PMID:28424574; For anti-RFP, please see reference PMID: 35705049.

Eukaryotic cell lines

Policy information about cell lines and Sex and Gender in Research

Cell line source(s)	HEK293T/17 cell line was purchased from ATCC (CRL-11268).
Authentication	The cell line was not authenticated.
Mycoplasma contamination	The cell line was periodically tested for mycoplasma, with no contamination detected.
Commonly misidentified lines (See ICLAC register)	No commonly misidentified cell lines were used in this study.

Animals and other research organisms

Policy information about studies involving animals; ARRIVE guidelines recommended for reporting animal research, and Sex and Gender in Research

Laboratory animals	VGAT-iCreER (C57BL/6N-Tg(Slc32a1-icre/ERT2)3Gloss/J, JAX 016582)<br>VGAT-Cre ($\text{Slc32a1}^{\text{tm2(cre)Lowl}}$/J, JAX 016962)<br>CMV-Cre (B6.C-Tg(CMV-cre)1Cgn/J, JAX 006054)<br>Scg2-tTA (B6.Cg-Tg(Scg2-tTA)1Jt/J, JAX 008284)<br>Ai93 (B6;129S6-$\textit{Igs7}^{\textit{tm93.1(tetO-GCaMP6f)Hze}}$/J, JAX 024103)<br>Ai85 (B6;129S-$\textit{Igs7}^{\textit{tm85.1(tetO-gltI/GFP*)Hze}}$/J, JAX 026260)<br>Rosa26-LSL-Cas9 (JAX 026175)<br><br>Mice of each sex of 8-12 weeks were used.
Wild animals	This study did not involve wild animals.
Reporting on sex	Mice of each sex (~130 males and ~130 females) were used in this study; we did not observe any effects of sex on the findings.
Field-collected samples	This study did not involve samples collected from the field.
Ethics oversight	All animal procedures were performed in accordance with the Guide for the Care and Use of Laboratory Animals as adopted and promulgated by the US National Institutes of Health. All procedures for testing and handling were approved by the Institutional Animal Care and Use Committee of Northwestern University.

Note that full information on the approval of the study protocol must also be provided in the manuscript.
